# Deep computational phenotyping of genomic variants impacting the SET domain of KMT2C reveal molecular mechanisms for their dysfunction

**DOI:** 10.3389/fgene.2023.1291307

**Published:** 2023-11-28

**Authors:** Salomão Dória Jorge, Young-In Chi, Jose Lizarraga Mazaba, Neshatul Haque, Jessica Wagenknecht, Brian C. Smith, Brian F. Volkman, Angela J. Mathison, Gwen Lomberk, Michael T. Zimmermann, Raul Urrutia

**Affiliations:** ^1^ Linda T. and John A. Mellowes Center for Genomic Sciences and Precision Medicine, Medical College of Wisconsin, Milwaukee, WI, United States; ^2^ Division of Research, Department of Surgery, Medical College of Wisconsin, Milwaukee, WI, United States; ^3^ Department of Biochemistry, Medical College of Wisconsin, Milwaukee, WI, United States; ^4^ Department of Pharmacology and Toxicology, Medical College of Wisconsin, Milwaukee, WI, United States; ^5^ Clinical and Translational Sciences Institute, Medical College of Wisconsin, Milwaukee, WI, United States

**Keywords:** genomic variants, H3K4 methyltransferase, Kmt2C, paralog annotation, SET domain

## Abstract

**Introduction:** Kleefstra Syndrome type 2 (KLEFS-2) is a genetic, neurodevelopmental disorder characterized by intellectual disability, infantile hypotonia, severe expressive language delay, and characteristic facial appearance, with a spectrum of other distinct clinical manifestations. Pathogenic mutations in the epigenetic modifier type 2 lysine methyltransferase KMT2C have been identified to be causative in KLEFS-2 individuals.

**Methods:** This work reports a translational genomic study that applies a multidimensional computational approach for deep variant phenotyping, combining conventional genomic analyses, advanced protein bioinformatics, computational biophysics, biochemistry, and biostatistics-based modeling. We use standard variant annotation, paralog annotation analyses, molecular mechanics, and molecular dynamics simulations to evaluate damaging scores and provide potential mechanisms underlying KMT2C variant dysfunction.

**Results:** We integrated data derived from the structure and dynamics of KMT2C to classify variants into SV (Structural Variant), DV (Dynamic Variant), SDV (Structural and Dynamic Variant), and VUS (Variant of Uncertain Significance). When compared with controls, these variants show values reflecting alterations in molecular fitness in both structure and dynamics.

**Discussion:** We demonstrate that our 3D models for KMT2C variants suggest distinct mechanisms that lead to their imbalance and are not predictable from sequence alone. Thus, the missense variants studied here cause destabilizing effects on KMT2C function by different biophysical and biochemical mechanisms which we adeptly describe. This new knowledge extends our understanding of how variations in the KMT2C gene cause the dysfunction of its methyltransferase enzyme product, thereby bearing significant biomedical relevance for carriers of KLEFS2-associated genomic mutations.

## 1 Introduction

The lysine methyltransferase 2 (KMT2) family (also known as MLL) contains important architectural proteins involved in gene regulation responsible primarily by methylation of histone 3 lysine 4 (H3K4), widely recognized as a mark of transcriptional activation. The post-translational modification (PTM) on K4 is dependent on which enzyme is involved in the catalysis, resulting in mono- (me1), di- (me2), and tri-methylation (me3) (Hu et al., 2013; Li et al., 2016; Bochyńska et al., 2018; Maurya et al., 2022).

**FIGURE 1:**
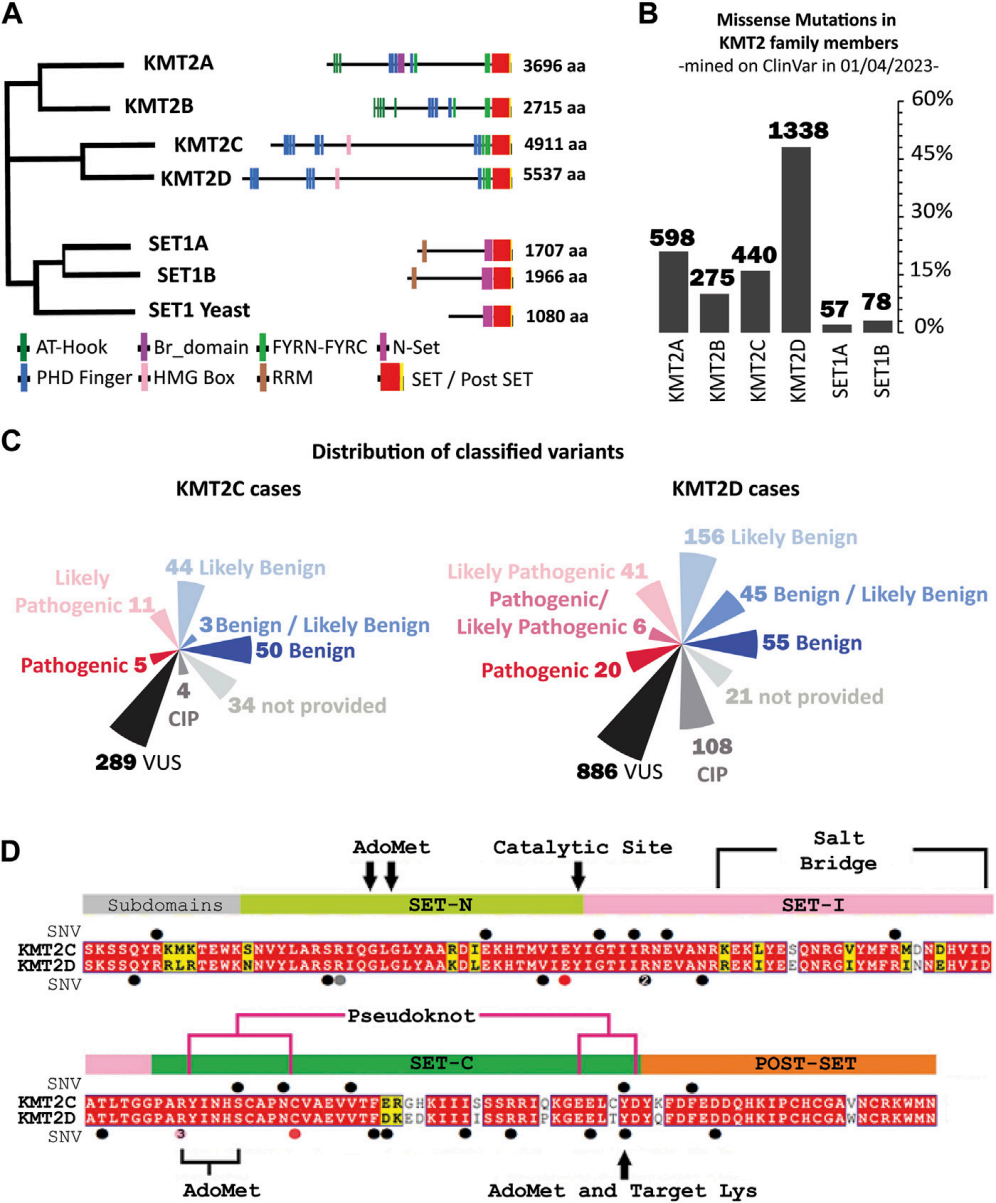
Sequence conservation and Variants in KMT2 family proteins: Evolution, Domains and Mutations. **(A)** The human KMT2 family is comprised of six members, which can be divided into three subclasses according to their evolutionary origins. (*left*) Evolutionary distances between KMT2 family protein sequences in Human and *Saccharomyces cerevisiae* (Set1 Yeast), generated by Clustal-Omega (Sievers et al., 2011) using the default settings, (*right*) Domain organization of the KMT2 proteins. The numbers indicate the number of amino acids. AT-Hook = adenosine-thymidine-hook; FYRN/FYRC = phenylalanine and tyrosine-rich region (*N*- and *C*-terminal); HMG, high mobility group; N-SET = N-terminal of SET; PHD, plant homeodomain; Post-SET, *C*-terminal of SET; RRM, RNA recognition motif; SET = Su(var)3–9. **(B)** Frequency of germline missense mutations across each member of the KMT2 family reveals that 48% of variants are attributed to the KMT2D gene, accounting for 1,338 entries. The other members of the family exhibited varying frequencies, with KMT2A having 598 (21.5%), KMT2C with 440 (15.8%), and KMT2B with 275 (9.9%). The remaining 5% of the population was represented by SETD1A (57 entries) and SETD1B (78 entries). **(C)** Current interpretation of the variants under study in ClinVar classified based on the five-classification system of KMT2C (*left*) and KMT2D (*right*). **(D)** SET domain multiple sequence alignment (MSA) of KMT2C (top) and KMT2D (bottom) paralogs. Positions that are identical between the orthologs are highlighted with a red background, and similar residues are written with bold black characters and boxed in yellow. Alignment was performed using the using the Align123 algorithm (HigginsThompson et al., 1994) available in Discovery Studio and displayed using Esprit 3.0 Server (Robert and Gouet, 2014). Relative positions of variants mined on ClinVar are labeled with secondary structure as circles on top of the KMT2C sequence and below the KMT2D sequence. Colors represent the clinical interpretation according to Figure 1C map (VUS as black circle, CIP as gray, likely pathogenic as pink, and pathogenic as red).

The human KMT2 family comprises six members, which can be divided into three subclasses according to their evolutionary origins, features, and functions (Figure 1A) (Crump and Milne, 2019; Sugeedha et al., 2021). KMT2A and KMT2B members are homologs of the Trithorax (Trx) gene, responsible for mono- and di-methylating H3K4 as promoters and with low levels of trimethylation activity also observed representing less than 5% of global H3K4me3 (Poreba et al., 2020). KMT2C and KMT2D members share the ancestor gene Trithorax-related (Trr) and are the principal genes responsible for the mono-methylation of H3K4 at enhancer regions. Lastly, SETD1A and SETD1B share the ancestor gene SET1 in yeast, and both members have distinct structural characteristics compared to the members mentioned above. SETD1B is functionally redundant to SETD1A in implementing H3K4me3 at highly expressed genes (Sze et al., 2020). The enzymatic activity of the KMT2 family is driven by a highly conserved 130–140 amino acid *C*-terminal catalytic Su(var)three to nine, Enhancer-of-zester and Trithorax, or SET, domain that methylate specific H3K4 sites by transferring a methyl group from the cofactor *S*-adenosyl methionine (SAM) (Dillon et al., 2005; Collins et al., 2019; Zheng et al., 2021).

Loss-of-function mutations in the KMT2 family of genes are implicated in various chromatinopathies, including Kleefstra Syndrome type-2 (KLEFS-2) and Kabuki Syndrome type-1 (KS-1). KLEFS-2 (OMIM 617768) is a rare genetic disorder associated with mutations in the KMT2C gene, characterized by individuals exhibiting intellectual disability, childhood hypotonia, autistic-like features, and distinctive facial features (Koemans et al., 2017; Lavery et al., 2020; Siano et al., 2022). Heterozygous loss of function mutations in the KMT2D gene (OMIM 147920) is the major primary cause of KS-1 and individuals who also manifest with intellectual disability and developmental delay and may share physical and behavioral characteristics with individuals with KLEFS-2 (Lavery et al., 2020; Barry et al., 2022).

Diagnosing rare diseases such as KLEFS-2 and KS-1 is challenging due to high clinical phenotypic heterogeneity (Siano et al., 2022). More information is needed to classify the genomic variants in the KMT2C gene, with nearly 80% of germline missense mutations lacking clear descriptions of their potential pathogenicity due to an insufficient understanding of the role of this gene in rare diseases. Therefore, in-depth investigations into the function and regulation of the KMT2C protein are necessary.

To further explore and elucidate the significance of genomic variants in KMT2C function, our study focused on variants within the SET catalytic domain. We applied the paralog annotation analysis (Ware et al., 2012; Walsh et al., 2014; DeVoe et al., 2021). This annotation method is a well-established methodology to identify and assess the impact of a single nucleotide variant on one member of evolutionarily related proteins by annotating the equivalent amino acid in the protein with unknown clinical genetic information (Walsh et al., 2014). For instance, if a missense mutation in the KMT2D gene located in chromosome 12q13 disrupts SET domain function in the KMT2D protein and results in KS-1, then a novel variant affecting the equivalent amino acid in KMT2C mapped in chromosome 7q36, located at a different chromosomal locus, would likely be pathogenic, causing KLEFS-2 in a patient.

In this study, we developed a multidimensional approach that includes standard variant annotation, paralog annotation, molecular mechanics scoring, and molecular dynamics simulations to understand the potential mechanisms by which the KMT2C variants can impact SET domain function. Combined, the data presented here provide valuable knowledge that aids in classifying genomic variants in the KMT2C gene and sheds light on the underlying molecular mechanisms. Furthermore, our findings may pave the way for future drug design by identifying druggable conformations specific to KMT2C mutations, thus offering potential therapeutic targets for ameliorating the symptoms of KLEFS-2. In conclusion, this new knowledge advances the field of chromatinopathies in rare and undiagnosed diseases.

## 2 Materials and methods

### 2.1 Data collection and variant pathogenicity classification

The germline missense variants were collected and annotated from ClinVar database (Landrum et al., 2016). Additionally, dbNSFP version 4.0 was used to annotated the missense mutations with 20 prediction algorithms including SIFT (Ng and Henikoff, 2003), SIFT4G (Vaser et al., 2016), PolyPhen2 (Adzhubei et al., 2010), FATHMM (Shihab et al., 2013), MutationAssessor (Reva et al., 2011), MutationTaster (Schwarz et al., 2014), MutPred (Li et al., 2009), PROVEAN (Choi and Chan, 2015), GERP_RS (Davydov et al., 2010), PhiloP30way (Pollard et al., 2010), PhastCons30way (Siepel et al., 2005), CADD (Rentzsch et al., 2019), DANN (Quang et al., 2014), fathmm_MKL (Shihab et al., 2015), fathmm_XF (Rogers et al., 2018), GenoCanyon (Lu et al., 2015), METALR (Sun and Yu, 2019), MetaSVM (Kim et al., 2017), REVEL (Ioannidis et al., 2016), and VEST4 (Carter et al., 2013). The genomic location, using the GRCh38 reference assembly, and altered base data for each variant were used as input in dbNSFP (Liu et al., 2020).

### 2.2 Modeling

The crystallized structure of the KMT2C in complex with cofactor product AdoHcy (SAH) and the H3 peptide, entry code 5F59 at 2.8 Å resolution (Li et al., 2016), were retrieved from RCSB Protein Data Bank (PDB) (Berman et al., 2007), and used as a geometric reference for constructing the biomacromolecule model. The missing loop (FEDDQH) connecting the N4889 and K4895 was built and optimized using the LOOPER algorithm (Spassov et al., 2008). The missense variants were built by substitutions into the wild-type (WT:KMT2C) protein, and side chain refinement was performed on mutated residues using ChiRotor algorithm (Spassov et al., 2007). Input conformations for MD simulations using the Discovery Studio suite version 21.1 (Dassault Systèmes BIOVIA) were obtained as follows. We first minimized the energy using Steepest Descent with 500 ps run at constant volume at 300K, followed by a Conjugate Gradient minimization with the Distance-Dependent Dielectrics solvent model with the same size and temperature. The structures of the energy-minimized models are available in Supplementary Table (KMT2C_PDBs.zip).

### 2.3 *In silico* mutagenesis and energy calculation

We evaluated the effect of single-point mutations in protein stability upon amino-acid substitution by calculating the change in folding free-energy (ΔΔG_folding_) using two different force field-based methods. CHARMM forcefield was used to perform the alanine scanning mutagenesis (ΔΔG_fold-ALAmut_) and the impact of variants on structural integrity (ΔΔG_fold-CHARMM_) using Calculate Mutation Energy (Stability) protocol in Discovery Studio (Adzhubei et al., 2010). Additionally, we evaluated the change in protein stability using FoldX (ΔΔG_fold-FOLDX_) for each variant (Shihab et al., 2013). The effect of a mutation on protein stability, ΔΔG_fold_, is calculated as the difference between the folding free energies, (ΔGfolding) of the mutant (ΔGfolding-mutant) and the wild type (ΔG_folding-WT_).

Folding energy values of ΔΔG_folding_ > 0.5 kcal/mol classify the effect of a variant as destabilizing, namely, unfolding propensity, whereas variants with values of ΔΔG_folding_ < −0.5 kcal/mol have a stabilizing effect, and variants with neutral effects yield values of ΔΔG_folding_ ranging from −0.5 to 0.5 kcal/mol.

*In silico* mutagenesis was performed using the Calculate Mutation Energy (Binding) protocol in Discovery Studio to assesses changes in the binding affinity of KMT2C:SAH and KMT2C:H3K4 peptide complexes in response to single-point mutations (Reva et al., 2011). The energy effect of each mutation on the binding affinity (mutation energy, ΔΔG_binding_) is calculated as the difference between the binding free energy in the mutated structure (ΔΔG_binding-mutant_) and wild type protein (ΔΔG_binding-mutant_). The binding free energy, ΔΔG_bind_, is defined as the difference between the free energy of the complex and unbound state. All energy terms are calculated by CHARMm and the electrostatics energy is calculated using a Generalized Born implicit solvent model.

### 2.4 Residual frustration analysis

To quantify changes in local interactions caused by each genomic variant, we used the algorithm developed by Ferreiro and Woylness (Ferreiro et al., 2007) for the calculation of residual frustration in the WT and variant structures. The single residual frustration index was calculated from the Frustratometer server (http://www.frustratometer.tk) (Parra et al., 2016). This server estimates the energy of a protein structure and compares it to the energies of a set of ∼1,000 decoy states.

The frustration index for the contact between the amino acids *i,j* was defined as a Z-score of the energy of the native pair compared to the N decoys. A Z-score >0.78 classifies the effect of a residue (or native contact) as minimally frustrated or stabilizing, whereas a residue (or native contact) with a Z-score < −1.0 classifies as highly frustrated or destabilizing, and residues (or native contacts) with neutral effects yield Z-scores ranging from −1.0 to 0.78 (Parra et al., 2016).

### 2.5 Molecular dynamics

MD simulations were performed using the CHARMM36 all-atom force field (Huang and MacKerell, 2013). The distance-dependent dielectrics were used as an implicit solvent model with a dielectric constant of 80 and a pH of 7.4. Each model was initially subjected to energy minimization for 5,000 steps using the steepest descent followed by 5,000 steps of conjugate gradient without any constraints/restraints on the KMT2C atoms. The system was then heated to 300 K at constant volume during 200 ps followed by 500 ps equilibration. The 10 ns production simulations were conducted under NPT thermodynamic ensemble with a step size of 2 fs. Trajectory files were recorded every 10,000 simulation steps to generate 1,000 conformations.

The trajectory files were analyzed for structural impact. The root mean square deviation (RMSD) was calculate including the protein backbone as reference. The root mean square fluctuation (RMSF) from the average structure in the trajectory was obtained by superimposition of each frame to the first frame before calculating the average structure. We also calculate the radius of gyration (Rg), solvent accessible surface area (SASA) and non-bond interactions analysis using the tools available within Discovery Studio. The data was plot in GraphPad Prism and Molecular visualizations were generated using PyMOL. The RMSF values have been written as B-factor in the models provided within the Supplementary Table (KMT2C_PDBs.zip).

### 2.6 MM/PBSA calculations

We performed the Molecular Mechanics Poisson-Boltzmann Surface Area (MM/PBSA) calculations to evaluate the binding affinities of KMT2C:complex interactions (Tirado-Rives and Jorgensen, 2006; Homeyer and Gohlke, 2012). The last 1,000 ps of the MD trajectories were analyzed at a 10 ps time interval to estimate the binding free energy (∆G_bind_), which was calculated by the following equation:

$$\Delta G_{\text{bind}}=G^{\text{complex}}\;–\;G^{\text{KMT}2C}\;–\;G^{\text{ligand}}$$ (1)

where G^complex^, G^KMT2C^ and G^ligand^ are the free energies of KMT2C:complexes; KMT2C (WT and variants) and ligand (SAH cofactor or H3 peptide) respectively. The Free Energy (G) of each state is estimated as follows:

$$G=E_{\text{MM}}+G_{\text{PB}}+G_{\text{SA}}\;–\text{TS}$$ (2)

$$E_{\text{MM}}=E_{\text{vdw}}+E_{\text{ele}}+E_{\text{bnd}}$$ (3)

where, in Eq. (2), E_MM_ is the molecular mechanical energy, G_PB_, and G_SA_ are the polar and non-polar contributions to the solvation-free energies, and TS is the entropic contribution of the system. E_MM_ was obtained by summing contributions from van der Waals, electrostatic, and bonded (bond, angle, and dihedral) interactions, according to Eq. (3).

Additionally, we performed calculations of interaction energy (CIE) to evaluate the nonbonded interactions (van der Waals term and the electrostatic term) between Zn^2+^ and KMT2C.

## 3 Results

### 3.1 Clinical significance of germline missense mutations in KMT2C and KMT2D genes

Genetic mutations are crucial in developing various diseases, including rare genetic disorders. Identifying and characterizing these mutations is essential for understanding disease mechanisms and improving patient care. Toward this end, we mined a comprehensive dataset of germline missense mutations in KMT2 family members from the ClinVar database.

We identified 2,786 germline missense mutations in KMT2 family members. Figure 1B illustrates the distribution of these mutations among different genes within the family. Approximately half of the variants (48%) are attributed to the KMT2D gene, accounting for 1,338 entries. The other members of the family exhibited varying frequencies, with KMT2A having 598 (21.5%), KMT2C with 440 (15.8%), and KMT2B with 275 (9.9%). The remaining 5% of the population was represented by SETD1A (57 entries) and SETD1B (78 entries).

To assess the clinical significance of the variants identified in KMT2C and KMT2D, we employed a five-classification system widely used in genetics research (Richards et al., 2015). This system classifies variants as Benign, Likely Benign, Variant of Uncertain Significance (VUS), Likely Pathogenic, and Pathogenic, based on their predicted impact on gene function. Figure 1C presents the classification distribution of the variants within KMT2C and KMT2D. Notably, most variants in both genes are classified as VUS. In KMT2C, 289 out of 440 variants (65.7%) fell into the VUS classification; in KMT2D, 886 out of 1,338 variants (66.2%) are classified as VUS. This underscores the challenges in determining the clinical implications of these variants due to their uncertain significance. Moreover, our analysis reveals a concerning number of conflicting interpretations. For instance, 0.9% of KMT2C (4 out of 440), and 8.1% of KMT2D (108 out of 1,338) variants were observed with conflicting interpretations, and 7.7% of KMT2C (34 out of 440) and 1.6% of KMT2D (21 out of 1,338) variants could not be classified.

Furthermore, a substantial percentage of germline missense variants in KMT2C (74.3%, 327 out of 440) and KMT2D (75.9%, 1,015 out of 1,338) cannot be confidently classified in terms of their potential pathogenicity. The high prevalence of VUS classifications, conflicting interpretations, and unclassified variants poses challenges in clinical decision-making and genetic counseling for patients and their families. These findings underscore the need for further research and functional studies to better understand the role of KMT2C and KMT2D genes in rare diseases. This led us to the current study that uses a multi-tier data-science approach to address this knowledge gap.

### 3.2 Classification of genomic variants in SET domains according to 2D sequence-based methods from clinical classification guidelines

The germline missense variants are distributed across the entire KMT2C and KMT2D proteins without a specific concentration bias, as depicted in Supplementary Figure S1 (Supplementary Material). However, the clinical implications of genomic variants within the SET domains of these proteins have garnered significant interest. In this study, we focused on the conserved sequence of 130–140 amino acids within the SET domain. KMT2C had 11 variants out of 440 while KMT2D had 20 variants out of 1,338 in this domain.

To identify the functionally equivalent amino acids across the KMT2C/D paralog proteins, we annotated the KMT2D variants by aligning the protein sequences (Figure 1D). The sequence identity for the full-length proteins was found to be 31.5% across the paralogs. When considering only the SET domain, the sequence identity was 81.2% among the paralogs. These findings highlight the conservation of the SET domains within the paralogous proteins, suggesting a shared functional role, despite variations in other regions of the proteins.

In this way, the total of 20 variants that were mined in KMT2D can then be used to annotate the equivalent amino acid in KMT2C, for which no clinical genetic information exists (Figure 1D; Table 1). The KMT2D variant D5489E was excluded from our analysis since the KMT2C analogous residue is already glutamic acid. There are two hotspots in KMT2D, one at position 5,471, with three variants submitted to Clinvar classified as likely pathogenic (R5471K, R5471S, and R5471W), and the other hotspot is at position 5,432, with two variants (R5432Q and R5432W). Paralog annotation revealed a third hot spot, with two variants with the same pattern of substitution of tyrosine by cysteine at position 4,884 in KMT2C and 5,510 in KMT2D.

**TABLE 1:** Germline missense and paralog annotated KMT2C variants mined on the SET Domain.

Gene	Variant	GRCh38^a^	Mutation	Gene(s)	Clinical data^b^	Clinical significance^c^	Paralog annotation^d^
**KMT2C**	R4763Q	7:152,144,768	c.14288 G > A	p.Arg4763Gln	NP	VUS	-
	E4792D	7:152,139,759	c.14376 G > C	p.Glu4792Asp	Kleefstra syndrome 2	VUS	-
	G4802R	7:152,139,731	c.14404 G > A	p.Gly4802Arg	NP	VUS	-
	I4805V	7:152,139,722	c.14413A > G	p.Ile4805Val	NP	VUS	-
	E4808K	7:152,139,713	c.14422 G > A	p.Glu4808Lys	NP	VUS	-
	R4828H	7:152,139,237	c.14483 G > A	p.Arg4828His	NP	VUS	-
	C4851Y	7:152,138,887	c.14552 G > A	p.Cys4851Tyr	NP	VUS	-
	N4854D	7:152,138,879	c.14560A > G	p.Asn4854Asp	NP	VUS	-
	V4860A	7:152,138,860	c.14579 T > C	p.Val4860Ala	NP	VUS	-
	Y4884C	7:152,136,917	c.14651A > G	p.Tyr4884Cys	NP	VUS	-
	F4890Y	7:152,136,899	c.14669 T > A	p.Phe4890Tyr	NP	VUS	
**KMT2D**	Q5387R	12:49,022,768	c.16160A > G	p.Gln5387Arg	NP	VUS	Q4761R
	S5404C	12:49,022,717	c.16211 C > G	p.Ser5404Cys	Kabuki syndrome 1	VUS	S4778C
	R5405H	12:49,022,714	c.16214 G > A	p.Arg5405His	NP	Likely benign	R4779H
	V5423F	12:49,022,661	c.16267 G > T	p.Val5423Phe	NP	VUS	V4797F
	E5425K	12:49,022,655	c.16273 G > A	p.Glu5425Lys	NP	Pathogenic	E4799K
	R5432Q	12:49,022,633	c.16295 G > A	p.Arg5432Gln	NP|Kabuki syndrome 1	CIP	R4806Q
	R5432W	12:49,022,634	c.16294 C > T	p.Arg5432Trp	NP	Pathogenic	R4806W
	N5437S	12:49,022,618	c.16310A > G	p.Asn5437Ser	NP	VUS	N4811S
	T5464M	12:49,022,301	c.16391 C > T	p.Thr5464Met	NP	VUS	T4838M
	R5471K	12:49,022,280	c.16412 G > A	p.Arg5471Lys	NP	Likely pathogenic	R4845K
	R5471S	12:49,022,151	c.16413 G > T	p.Arg5471Ser	Kabuki syndrome 1	Likely pathogenic	R4845S
	R5471W	12:49,022,281	c.16411A > T	p.Arg5471Trp	Kabuki syndrome 1	Likely pathogenic	R4845W
	C5481Y	12:49,022,122	c.16442 G > A	p.Cys5481Tyr	Kabuki syndrome|NP	P/LP	C4855Y
	F5488L	12:49,022,100	c.16464 T > A	p.Phe5488Leu	NP	VUS	F4862L
	D5489E	12:4,9,022,097	c.16467 C > G	p.Asp5489Glu	Kabuki syndrome	VUS	D4863E^e^
	I5496S	12:4,9,022,077	c.16487 T > G	p.Ile5496Ser	NP	VUS	I4870S
	R5500W	12:4,9,022,066	c.16498 C > T	p.Arg5500Trp	NP	VUS	R4874W
	E5507D	12:4,9,022,043	c.16521 G > C	p.Glu5507Asp	NP	VUS	E4881D
	Y5510C	12:4,9,021,865	c.16529A > G	p.Tyr5510Cys	Neurodevelopmental disorder	VUS	Y4884C
	D5518G	12:4,9,021,841	c.16553A > G	p.Asp5518Gly	Kabuki syndrome	VUS	D4892G

^a^ Chromossome:Location.

^b^ NP: not provided.

^c^ VUS: variant of uncertain significance; CIP: conflicting interpretations of pathogenicity; P/LP: Pathogenic/Likely Pathogenic.

^d^ Annotated equivalent amino acid in KMT2C.

^e^ Variant excluded from analysis since the KMT2C analogous residue is already glutamic acid.

**FIGURE 2:**
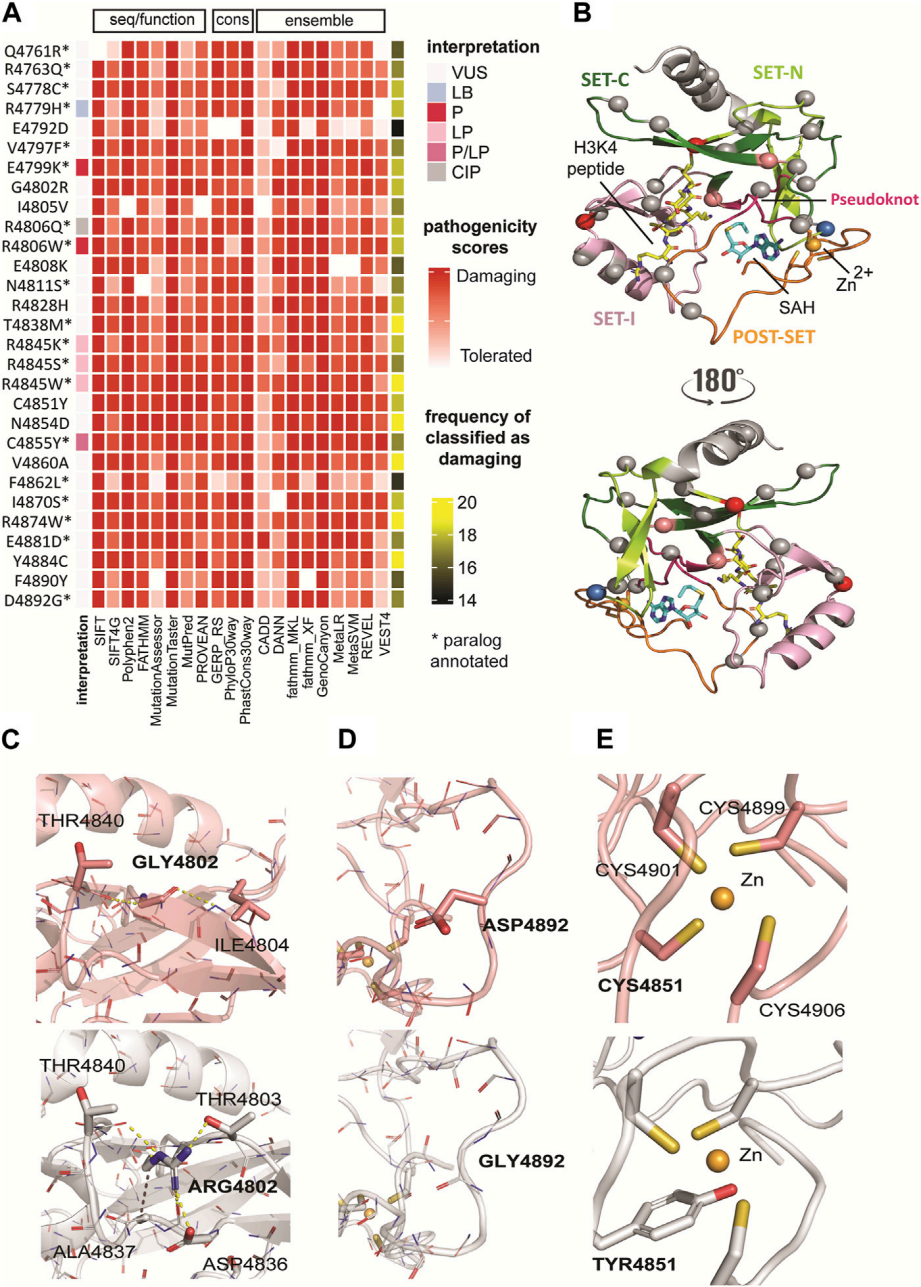
Classification of genomic variants according to sequence- and structure-based methods. **(A)** Classification of genomic variants in SET domain based on sequence/functional, ensemble, and evolutionary/conservation scoring methods. The pathogenicity scores from 20 selected tools showed a consensus result for variants T4838M, R4845W, N4854D, V4860A, R4874W, and Y4884C. All scores classified these variants as damaging. Based on the cumulative analysis, variant E4792D showed a lower frequency (74%), with 15 of 20 tools predicting it as deleterious. **(B)** Mapping of paralog missense variants onto the KMT2C SET domain structure in complex with cofactor product SAH and the histone H3K4 peptide (PDB ID: 5F59 at 2.80 Å resolution)^2^. The same color codes for the sub-domains shown in Figure 1D are used. The protein is shown in cartoon representation. The cofactor product SAH and the substrate H3K4 are shown in a stick model with carbon atoms colored in blue and yellow, respectively, oxygen in red, nitrogen in blue, and sulfur in yellow. The Zn2+ ion is shown as an orange sphere. Mapped variants are shown as spheres and colored according ClinVar interpretation indicated in the legend in Figure 2A. **(C)** The G4802R variant showed the highest destabilizing effect. The substitution of Gly by Arg with bulky and positive side chain, results in partial loss of secondary structure of the third beta-sheet due to unfavorable steric clashes and polar solvation energy. **(D)** The D4892G variant has stabilizing effect. This substitution between the last two loops at the C-terminus nullifies the effect of the side chain in the same manner as alanine mutagenesis. However, unlike alanine, glycine maintains the conformational flexibility of the backbone, resulting in a favorable effect for this substitution and this protein region. **(E)** The C4891Y variant disrupts the Zn2+ coordination because of inability of Tyr to make coordinate bond and the steric clashes introduced upon substitution. WT (top) and variant (bottom) structures are colored in salmon and gray, respectively. Polar interactions are represented as yellow dashed lines and hydrophobic bonds in purple. The backbone atoms are shown in line model, and the sidechain atoms as stick models.

As previously noted, out of 31 variants annotated in the SET domain, 23 (74.2%) were classified as VUS, which is highly problematic since they are in the catalytic methyltransferase domain. Therefore, the precise classification of the variants is fundamental for a better understanding of the functional mechanism of the protein. To enhance the interpretation and the prediction of variants with functional consequences, we started scoring the germline variants within the SET domain with the sequence-based methods (2D). We used available pathogenicity prediction algorithms based on chromosome and position on genome version GRCh38. Scouring the dbNSFP4.0 database, we obtained unsupervised scores relevant to coding variants. The threshold levels to classify variants into benign/tolerant/neutral and damaging/deleterious categories are described in Supplementary Table S1 (Supplementary Material). The lack of consensus in the classification is due to the different considerations and implications of each score (Figure 2A), as they vary from sequence conservation and machine learning to evolutionary constraints. Due to limitations in functional data, understanding how the different scoring methods are put together is imperative. Each scoring algorithm is based on different metrics. Briefly, sequence/functional scoring methods are based on the structure and the function of human proteins; ensemble scores are based on machine learning techniques and predictions; and evolutionary/conservation scores are based on observed and expected mutation rates (Panchenko et al., 2004).

The pathogenicity scores from 19 selected tools showed a consensus result for KMT2C variants T4838M, R4845W, N4854D, V4860A, R4874W, and Y4884C. All scores classified these variants as damaging. Based on the cumulative analysis, variant E4792D showed a lower frequency (75%), with 15 of 20 tools predicting it as deleterious. These tools often have conflicting predictions and vary in accuracy and reliability, which prompted us to investigate these variants further using 3D structure-based analyses.

### 3.3 Classification of genomic variants in SET domains according to structure-based methods

We further investigated the potential damage caused by genomic mutations on the protein structure. Based on multiple sequence alignment among the paralogues, we used the crystal structure of the KMT2C SET domain in complex with the cofactor product AdoHcy (*S*-adenosyl homocysteine, SAH) and the H3K4 peptide (PDB ID 5F59 at 2.8 Å resolution) (Li et al., 2016) as the template for homology modeling (see Methods for complete model building). Additionally, this template includes the *C*-terminal post-SET domain (region 4,886–4,911) where Zn^2+^ coordinates with the three cysteines (C4899, C4901, and C4906) together with a fourth cysteine (C4851) close to the SET domain active site (Figure 2B). Subsequently, we generated models for pathogenic variants using optimized *in silico* mutagenesis methods (Wells, 1991; Mishra et al., 2012).

We first performed alanine scanning mutagenesis to determine the contribution of amino acid side chains to KMT2C protein stability. For each variant, the simulation calculates the difference between the energy that is either favorable or unfavorable to protein folding (folding free energy, ΔΔG_fold-ALAmut_) of the wild type and variants. The results reported in Table 2, show that most residues (23 of 30) have neutral effects in stability when substituted by alanine.

**TABLE 2:** Scores and classification of KMT2C variants in the SET domain based on structural methods.

*Mutation Energy - Stability*							*Mutation Energy - Binding*				*Frustratometer Index*	
Variant	**ΔΔG**_**fold-ALAmut**_	*Effect^a^*	**ΔΔG**_**fold-FOLDX**_	*Effect^a^*	**ΔΔG**_**fold-CHARMM**_	*Effect^a^*	**ΔΔG**_**binding-H3K4**_	*Effect^a^*	**ΔΔG**_**binding-SAH**_	*Effect^a^*	**ΔF**	*Effect^b^*
Q4761R	0.0	Neutral	−0.78	Stabilizing	−0.12	Neutral	0.12	Neutral	0.08	Neutral	−0.07	Neutral
R4763Q	−0.2	Neutral	−0.64	Stabilizing	−0.02	Neutral	−0.12	Neutral	−0.05	Neutral	0.48	Neutral
S4778C	−0.2	Neutral	5.40	Destabilizing	1.24	Destabilizing	−0.06	Neutral	−0.04	Neutral	1.69	Minimally Frustrated
R4779H	−0.6	Stabilizing	0.21	Neutral	0.91	Destabilizing	−0.18	Neutral	−0.26	Neutral	−0.71	Neutral
E4792D	0.2	Neutral	−0.35	Neutral	0.93	Destabilizing	−0.01	Neutral	0	Neutral	0.64	Neutral
V4797F	0.0	Neutral	8.89	Destabilizing	−0.13	Neutral	−0.03	Neutral	−0.01	Neutral	0.32	Neutral
E4799K	0.3	Neutral	2.04	Destabilizing	4.13	Destabilizing	0.45	Neutral	0.1	Neutral	−0.29	Neutral
G4802R	0.0	Neutral	31.81	Destabilizing	12.37	Destabilizing	0.97	Destabilizing	−0.03	Neutral	−0.18	Neutral
I4805V	0.1	Neutral	1.03	Destabilizing	1.25	Destabilizing	0.08	Neutral	0	Neutral	−0.24	Neutral
R4806Q	−0.3	Neutral	−0.13	Neutral	−0.59	Stabilizing	−0.21	Neutral	−0.04	Neutral	−1.62	Highly Frustrated
R4806W	−0.3	Neutral	0.41	Neutral	−1.15	Stabilizing	−0.19	Neutral	−0.04	Neutral	−0.94	Neutral
E4808K	0.6	Destabilizing	1.13	Destabilizing	1.04	Destabilizing	0.77	Destabilizing	0.11	Neutral	−1.70	Highly Frustrated
N4811S	0.4	Neutral	0.23	Neutral	0.8	Destabilizing	0.71	Destabilizing	0.01	Neutral	0.02	Neutral
R4828H	−0.2	Neutral	6.06	Destabilizing	1.17	Destabilizing	0.94	Destabilizing	−0.1	Neutral	−0.42	Neutral
T4838M	−0.1	Neutral	0.62	Destabilizing	0.91	Destabilizing	−0.21	Neutral	−0.03	Neutral	−0.72	Neutral
R4845K	−0.2	Neutral	−0.86	Stabilizing	1.61	Destabilizing	−0.2	Neutral	0.49	Neutral	−0.40	Neutral
R4845S	−0.2	Neutral	2.31	Destabilizing	2.33	Destabilizing	−0.34	Neutral	0.22	Neutral	0.52	Neutral
R4845W	−0.2	Neutral	6.29	Destabilizing	0.88	Destabilizing	−0.72	Stabilizing	0.4	Neutral	1.91	Neutral
C4851Y	4.6	Destabilizing	9.23	Destabilizing	3.46	Destabilizing	0.23	Neutral	4.9	Destabilizing	−1.85	Highly Frustrated
N4854D	0.0	Neutral	2.23	Destabilizing	1.76	Destabilizing	−0.2	Neutral	−0.2	Neutral	−0.18	Neutral
C4855Y	0.0	Neutral	5.52	Destabilizing	−0.93	Stabilizing	0.01	Neutral	−0.01	Neutral	−0.72	Neutral
V4860A	0.0	Neutral	2.77	Destabilizing	1.35	Destabilizing	0.01	Neutral	0	Neutral	−0.98	Neutral
F4862L	0.0	Neutral	1.56	Destabilizing	1.28	Destabilizing	0.02	Neutral	0	Neutral	1.91	Minimally Frustrated
I4870S	0.1	Neutral	5.99	Destabilizing	3.96	Destabilizing	0.07	Neutral	0.01	Neutral	−2.15	Highly Frustrated
R4874W	−0.4	Neutral	1.84	Destabilizing	0.75	Destabilizing	−0.17	Neutral	−0.2	Neutral	−2.45	Highly Frustrated
E4881D	0.3	Neutral	3.18	Destabilizing	3.05	Destabilizing	0.03	Neutral	−0.01	Neutral	0.44	Neutral
Y4884C	0.8	Destabilizing	2.65	Destabilizing	3.47	Destabilizing	0.76	Destabilizing	0	Neutral	0.92	Minimally Frustrated
F4890Y	1.1	Destabilizing	0.44	Neutral	−0.59	Stabilizing	−0.17	Neutral	−0.01	Neutral	−2.26	Highly Frustrated
D4892G	0.5	Destabilizing	−1.17	Stabilizing	−1.3	Stabilizing	0.38	Neutral	0.14	Neutral	0.69	Neutral

^a^ Values of ΔΔGfolding >0.5 kcal/mol classifies the effect of variant as destabilizing, namely, unfolding propensity, whereas variants with values of ΔΔGfolding < −0.5 kcal/mol have stabilizing effect, and variants with neutral effects yield values of ΔΔGfolding ranging from −0.5 to 0.5 kcal/mol.

^b^ Values of Z-score >0.78 classifies the effect of residue (or native contact) as minimally frustrated or stabilizing, whereas residue (or native contact) with values of Z-score < −1.0 classifies as highly frustrated or destabilizing, and residues (contacts) with neutral effects yield values of Z-score ranging from −1.0 to 0.78.

We also highlight the destabilizing effect caused by mutating the side chain of C4851, a residue that contributes to Zn^2+^ binding via thiol interaction (Zhang et al., 2003). The alanine substitution at this position displays a variation in folding energy (ΔΔG_fold-ALAmut_ (C4851A) = 4.63 kcal/mol) caused by a loss of interaction. We report the combined results from alanine scanning mutagenesis for each amino acid within the SET domain in Supplementary Figure S2 (Supplementary Material).

We also used other methods for calculating the impact of variants on structural integrity, namely, thermodynamic stability (ΔΔG_folding_) of variants. We leveraged two different force field-based methods, CHARMM (ΔΔG_fold-CHARMM_) (Brooks et al., 2009) and FoldX (ΔΔG_fold-FoldX_) (Schymkowitz et al., 2005), respectively, for fast computational mutagenesis. Through this analysis, we found two structure-based scores with a high degree of agreement across the variants (Table 2), reflecting more variants with destabilizing effects. For example, the G4802R variant showed the highest destabilizing effect by both methods (ΔΔG_fold-CHARMM_ = 12.37 kcal/mol, ΔΔG_fold-FoldX_ = 31.82 kcal/mol) among all variants. The substitution is non-conservative and replaces Gly with bulky and positive side chain, resulting in partial loss of secondary structure of the third beta-sheet due to unfavorable steric clashes and polar solvation energy (Figure 2C). In contrast, the D4892G variant has stabilizing effect calculated by both methods (ΔΔG folding-CHARMM = −1.3 kcal/mol, ΔΔG folding-FOLDX = −1.17 kcal/mol). This substitution between the last two loops at the *C*-terminus nullifies the effect of the side chain in the same manner as alanine mutagenesis. However, unlike alanine, glycine maintains the conformational flexibility of the backbone, resulting in a favorable effect for this substitution and this protein region (Figure 2D).

We also calculated the effect of missense mutations in KMT2C cofactor and substrate complexes to identify the differences in the binding (free energy of binding) between the wild-type and mutated structures. In the KMT2C:SAH complex, only the variant C4851Y has a destabilizing effect. This missense mutation disrupts the Zn^2+^ coordination because of the inability of Tyr to make coordinate bond and the steric clashes introduced upon substitution (Figure 2E). Further, it displaces the residue H4900 enough to lose the strong N-H main-chain bond with the SAH-N1-Adenine moiety, resulting in a value of ΔΔG_binding-SAH_ = 4.9 kcal/mol. In the KMT2C:H3K4 peptide complex, five variants show destabilizing effects. The polar and positive residues in variants G4802R and E4808K change the electrostatic properties by forming additional intramolecular short-range interactions that affect the H3K4 binding site. Substitutions in variants N4811S, R4828H, and Y4884C cause a loss of interactions with the peptide due to direct changes in the binding site. The two-dimensional interactions of variants that destabilize the KMT2C are reported in Supplementary Figure S3 (Supplementary Material).

Finally, we studied how each genomic variant changes the local interactions by calculating the protein`s residual frustration (F) (Parra et al., 2016). Protein frustration refers to the suboptimal or energetically unfavorable interactions locally within a protein, which might differ between WT and damaging mutations. The variations (ΔF) in the configurational frustration index caused by a mutation (Fmut) can quantify a tendency to cause a conformational change in the protein (Dixit and Verkhivker, 2011). The configurational frustration indices calculated through single residual frustration (F_wt_) analysis indicates that the KMT2C SET domain is slightly lower in frustration index than a typical globular protein (Ferreiro et al., 2007). Using the cutoff values of frustration indices (see Methods), 7% of residues are highly frustrated (compared with 10% observed in a typical protein), and 34% of the residues are minimally frustrated (40% in a typical protein). The frustration values range between −1.92 and +2.25. After calculating the ΔF values (ΔF = Fmut - Fwt), we observed that substitutions in six genomic variants (R4806Q, E4808K, C4851Y, I4870W, R4874W, and F4890Y) affect the protein stabilization as the mutant residues are highly frustrated (ΔF < −1). Thus, the structural information obtained from thermodynamic parameters reveals that all genomic variants, except for Q4761R, R4763Q, and R4806W, cause significant perturbation in the folding and binding energies.

### 3.4 Classification of genomic variants in SET domains according to dynamic-based methods

We performed molecular dynamic simulations to study the behavior of normal and mutated KMT2C. For this, we evaluated protein stability using several local geometrical features, including root mean square deviation (RMSD), root mean square fluctuation (RMSF), the radius of Gyration (Rg), and solvent accessible surface area (SASA). To better characterized the difference between the WT:complex and the genomic variant, we used the average difference over ten replicates.

To document how these complexes change over time and whether they stabilize, we analyzed the energy variations of the normal KMT2C complexes during the simulations. Supplementary Figure S4 (Supplementary Material) shows the changes in total energy for each simulation replicate after 10 nanoseconds (ns) of simulation. The protein stabilizes at approximately 4 ns, with repetitive movements. The energy of the system reached the region of −7,570 kcal/mol, indicating a more stable state of this protein. To further analyze the time-dependent changes of the molecular interactions, we selected 250 conformations from the last 2.5 ns of each simulation. These conformations represent different snapshots of the protein during the simulation period.

**FIGURE 3:**
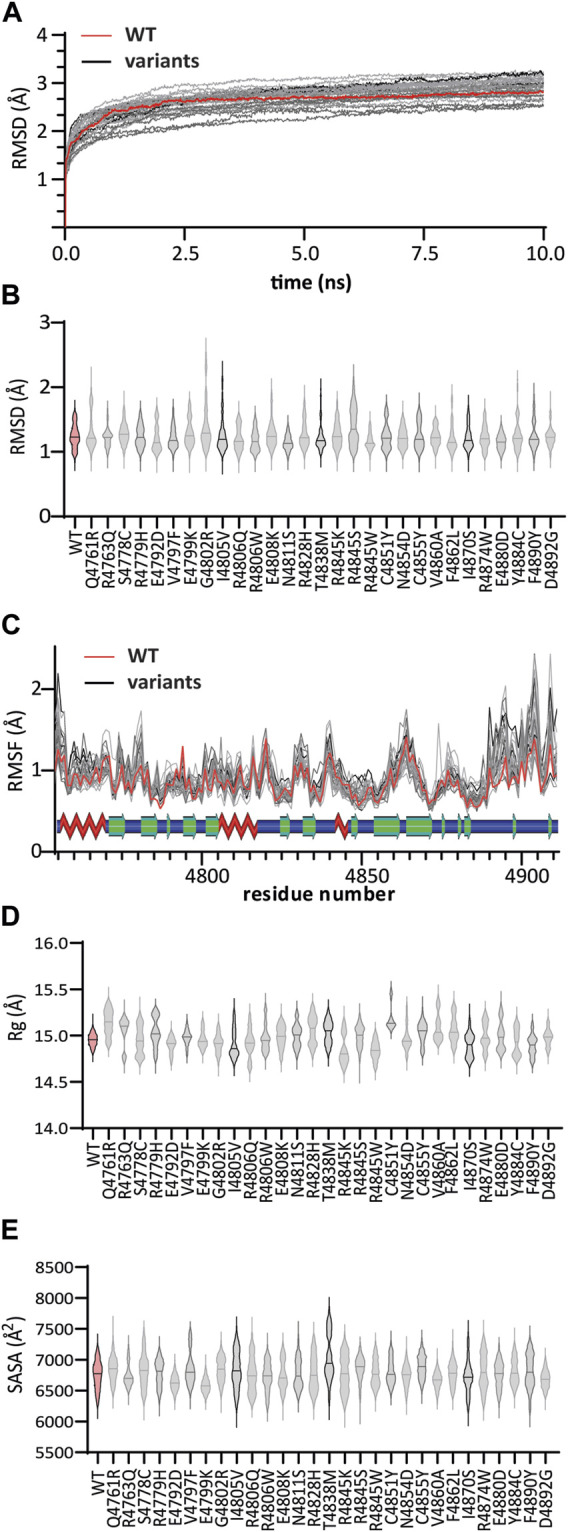
MD trajectory analyses of WT:KMT2C (red) and variant (gray) complexes: **(A)** Root mean square deviations (RMSD) from the corresponding initial structure emphasize the structure stability of WT:KMT2C during the simulation period consistent values from 7.5 to 10 ns **(B)** violin plot of backbone RMSD values, showing the median (solid lines) inside the kernel density plots. Among all variants, the G4802R variant showed higher values throughout simulations ranging from 0.95 Å to 2.65 Å, indicating that the G4802R variant is more unstable than the WT. **(C)** The plotted Root Mean Square Fluctuations identified two major regions, around residues 4,770 to 4,785 and 4,889 to 4,902, where all genomic variants have higher fluctuation values for individual residues than WT. On the other hand, RMSF values show a loss of flexibility, for all variants, at a region composed of the β6 and β7 sheet and conserved first knot region, as observed by lower RMSF values. The secondary structure elements are shown above, with beta sheets as green arrows, helices as red zig-zags, and loops as blue bars. **(D)** The Radius of Gyration (Rg) for the WT complex shows values that range between 14.7 and 15.2 which reflex a stable folded protein and compactness during the simulation. Except for the variant E4792D, with relatively similar ranging values (14.7 – 15.1), the remainder displayed higher variation in folding (unstable folding) compared to WT. **(E)** Solvent accessibility surface area (SASA) assesses significant changes in the surface exposure of native hydrophobic regions. Compared with the WT structure with a SASA of 6,756 ± 230 Å^2^, variants R4763Q, R4806Q, E4808K, N4854D, and E4880D did not change significantly. In contrast, a significant decrease in average SASA was observed for the E4792D, E4799K, R4806W, V4860A, I4870S, and D4892G variants. The remaining 18 variants showed an increase in SASA values, resulting in a decrease in the overall compactness and stability of the protein. Panels **(D)** and **(E)** plots follow the same pattern as **(B)**.

The root-mean-square deviations (RMSD) of backbone atoms, Figure 3A, also emphasize the structure stability of WT:KMT2C during the simulation period. The RMSD values from WT:KMT2C, highlighted as a red line, showed consistent values from 7.5 to 10 ns. Hence, these results indicate that WT:KMT2C remained stable throughout the simulation period. The consistent dynamics and limited variation in energy reflects the maintenance of conformational equilibrium. Congruently, the RMSD values of the backbone atoms further support structural stability. Thus, combined, our computer simulations lend insights into the behavior of this protein, which maintains stability over time. This information contributes to our understanding of the structural dynamics of KMT2C and its functional implications.

The RMSD is a popular metric for the assessment of structural similarity in 3D-structure by measuring the distance between the protein atoms of two structures during the simulation periods (Maiorov and Crippen, 1994). Higher deviation occurs across dissimilar structures, reflective of an unstable protein, whereas values near zero indicate identical conformation structures. Among all variants, the G4802R variant showed higher RMSD values throughout simulations with RMSD values ranging from 0.95 Å to 2.65 Å, indicating that the G4802R variant is more unstable than the WT (Figure 3B).

To complement the results of RMSD, we compared the local protein flexibility of the WT and the variants using RMSF values. The results plotted in Figure 3C show fluctuation values for individual residues and provide evidence of a change in local conformational dynamics of variants compared to WT. Notably, we identified two major regions, around residues 4,770 to 4,785 and 4,889 to 4,902, where all genomic variants have higher RMSF values than WT. The first region, located between β1 and β2 strands, is involved in interactions with the SAM cofactor, and the second region, located at the post-SET loop, links the SET-C region with the conserved post-SET Zn^2+^-tetrathiolate. On the other hand, RMSF values show a loss of flexibility, for all variants, at a region composed of the β6 and β7 sheet and conserved first knot region, as observed by lower RMSF values. This observation suggests that a high RMSF related to the alignment of these two parallel loops is vital to maintain the integrity of the substrate groove. Nevertheless, these differences in RMSF provide an image of how the variants can affect the binding mode of the SAM cofactor and the substrate recognition.

Based on previous work from our group (Chi et al., 2021; Chi et al., 2022), we used two different RMSF scores, namely,: Pearson correlation coefficient score (r-RMSF) and the average of absolute difference score (Δ|RMSF|). The complete RMSF profile of the WT and variants are shown in Supplementary Material (Supplementary Figure S5), and the scores are listed in Supplementary Table S2.

The variation of Rg values measures the degree of compactness and folding of a protein in relation to a circle or, in this case, a sphere across the simulation period. For the WT complex, we find values that range between 14.7 and 15.2 (Figure 3D), which reflex a stable folded protein and compactness during the simulation. Except for the variant E4792D, with relatively similar ranging values (14.7 – 15.1), the remainder displayed higher variation in folding (unstable folding) during the simulation period compared to WT.

Finally, we computed the SASA of the normal and variant KMT2C systems. SASA helps us understand changes in exposed hydrophobic regions of proteins. Hydrophobic regions are typically buried in the interior of proteins, which mostly have charged surfaces to contact solvent molecules. Consequently, SASA analysis assesses significant changes in the surface exposure of native hydrophobic regions between the normal and variant KMT2C systems, which reflects structural alterations (Ausaf Ali et al., 2014). Compared with the WT structure with a SASA of 6,756 ± 230 Å^2^, variants R4763Q, R4806Q, E4808K, N4854D, and E4880D did not change significantly. In contrast, a significant decrease in average SASA was observed for the E4792D, E4799K, R4806W, V4860A, I4870S, and D4892G variants (Figure 3E). The remaining 18 variants showed an increase in SASA values, resulting in a decrease in the overall compactness and stability of the protein. Therefore, combining these four different structural and dynamic metrics reflects the proper folding and dynamic stability of the genomic variants studied.

### 3.5 Genomic variants affect the time-dependent interaction with essential functional cofactors

To assess the impact that each genomic variant has on its affinity for its SAM cofactor and histone substrate, we used the molecular mechanics-Poisson-Boltzmann Surface Area (MM-PBSA) technique, which calculates binding energies for the formation KMT2C:SAH (ΔΔG_MMPBSA-SAH_) and KMT2C:H3K4 (ΔΔG_MMPBSA-H3K4_) complexes respectively. This method highly correlates with *in vitro* binding affinity measured experimentally for several systems (Genheden and Ryde, 2015; Wang et al., 2017).

The binding energy was calculated by averaging 150 snapshots extracted from MD conformational sampling for each replicate. The binding energy change for each variant, denoted as ΔΔG_bind_, was then obtained by subtracting the binding energy from the WT complex. Hence, a negative ΔΔG_bind_ value indicates improved binding affinity with KMT2C, whereas positive ΔΔG_bind_ values imply weaker binding affinity.

**FIGURE 4:**
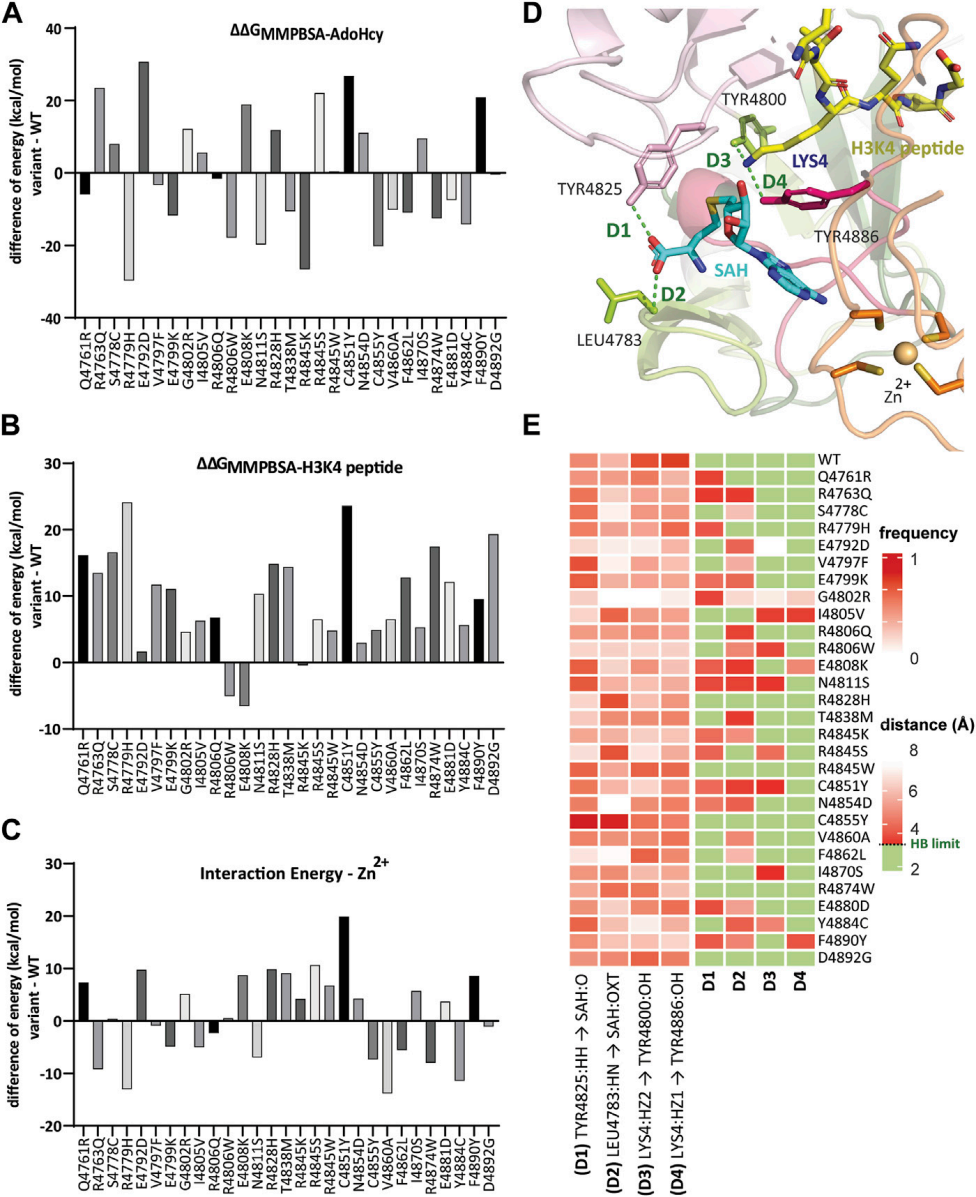
Genomic variants affect the time-dependent interaction with essential functional cofactors. Bar graph illustrating the binding free energy difference calculated from MM-PBSA of WT:KMT2C and variants in complex with **(A)** SAH and **(B)** H3K4 peptide calculated by averaging 150 snapshots extracted from MD conformational sampling for each replicate. The binding energy change for each variant, denoted as ΔΔG_bind_, was then obtained by subtracting the binding energy from the WT complex. Hence, a negative ΔΔG_bind_ value indicates improved binding affinity with KMT2C, whereas positive ΔΔG_bind_ values imply weaker binding affinity. **(C)** Zn^2+^ interaction energy differences showed the SNV in the conserved Zn^2+^-tetrathiolate at position 4,851 (C4851Y) has the weakest interaction with decrease of 19.9 kcal/mol compared to WT. **(D)** Mapped hydrogen bond interactions between conserved residues Tyr4825 and Leu4783 with SAH, and Tyr4800 and Tyr4886 with H3K4 peptide, showed in green dashed lines. The protein residues are shown as a stick model. **(E)** (*left*) Time-dependent non-bond monitoring at the last quarter of MD simulations is displayed as a heatmap (scale 0 to 1, where frequency = 1 represents the specific bond detected on 100% of conformations throughout the MD trajectory). The partners in the interaction are From. atom - To. atom. Greek letter locants are used to identify the relative location of atoms (A = alpha, B = beta, G = gamma, and D = delta). (*right*) Distances between the monitored bonds. We defined hydrogen bonds ranging from 2.2 to 3.3 Å filled in green color.

Compared with the WT:KMT2C (ΔΔG_MMPBSA-SAH_ = −163.4 kcal/mol), only variants R4806Q, R4845W, and D4892G show similar ΔΔG_AVERAGE_, with differences ranging within 2 kcal/mol (Figure 4A). Twelve variant models display decreased binding affinity for SAH. The variants R4763Q, E4792D, E4808K, R4845S, C4851Y, and F4890Y with ΔΔG_AVERAGE_ values ranging from −144.9 to −132.8 kcal/mol, respectively, demonstrate the lowest binding energies, reflecting a decrease in binding affinity of more than 12% compared with WT.

In contrast, increased binding affinity was observed for 14 variants with ΔΔG_AVERAGE_ values ranging from −169.3 to −193.1 kcal/mol. Only the R4806W variant from this group of 14 also showed an increased binding affinity to H3K4, with ΔΔG_MMPBSA-H3K4_ = −103.1 kcal/mol, compared to WT (ΔΔG_MMPBSA-H3K4_ = −98.0 kcal/mol). In total, as shown in Figure 4B, only two more variants show negative values, variant E4808K with ΔΔG_MMPBSA-H3K4_ = −104.6 kcal/mol, and variant R4845K with similar ΔΔG_MMPBSA-H3K4_ = −98.5 kcal/mol. The remaining 26 variants display binding affinity to H3K4 with lower binding energy values than WT. It is important to highlight the loss of 25% of binding affinity by variants R4779H and C4851Y, with ΔΔG_MMPBSA-H3K4_ = −74.5 and −73.9 kcal/mol, respectively. Both variants also exhibit the highest ΔΔG_bind_ to SAH, ΔΔG_bind-R4779H_ = −29.7 kcal/mol and ΔΔG_bind-C4851Y_ = 26.7 kcal/mol, reflecting the dynamics of adjacent pockets and the importance of the interactions between SAH and H3K4 pockets sites.

We extended our studies with a second approach for calculating interaction energy (CIE) between Zn^2+^ and the protein based on Van der Waals (VdW) and electrostatic interactions. This critical regulatory mechanism involves metal binding to the post-SET disordered region and can induce a disorder-to-order transition (Martin and Zhang, 2005; Selevsek et al., 2009). The SNV in the conserved Zn^2+^-tetrathiolate at position 4,851 (C4851Y) showed a lower interaction energy to Zn^2+^, with a 19.9 kcal/mol decrease compared to WT (Figure 4C). Altogether 14 variants show decreased binding energies, while the other 14 had increased values. Supplementary Table S2 (Supplementary Material) provides a complete description of these results.

Thus, this approach further provides insight into how changes in protein structure affect the dynamics of interactions among the regions of KMT2C and influence the recognition and binding affinity of the SAM cofactor, histone substrate, and Zn^2+^.

### 3.6 Genomic variants affect bond formation by conserved residues within the SAH-binding pocket and in the catalytic site

The fitness of variants for ligand binding was determined by the MM-PBSA method. This computational approach estimates the binding between a protein and other molecules (measured in binding free energy terms) (Srinivasan et al., 1998; Kollman et al., 2000). By computing binding free energy values, we assess stability and affinity of protein-ligand complexes, which is crucial to understand the impact of mutations on protein-ligand interactions.

We mapped four critical conserved residues in the SAH cofactor binding pocket and H3K4 binding channel during the last quarter of MD simulations (Figure 4D). We created two heatmaps to better understand the time-dependent hydrogen bond interactions and monitor the distances throughout the trajectory (Figure 4E). In our analysis, hydrogen bonds are identified to be formed with an upper limit set at 3.3 Å for the distance between the donor and acceptor atoms. These results demonstrated that there are variants where the frequency of interaction increase between residues Tyr4825 (D1) and Leu4783 (D2) with SAH when compared to WT; however, when analyzed together with the distance map, measuring the quality of these interactions is possible. Except for variants R4845W and C4855Y, all variants showed increased distances, which defines this contact as a weak dipole-dipole interaction. In contrast, all variants showed a decrease in the frequency of hydrogen bonding between the conserved Tyr4800 and Tyr4886 with the Lys4. These two amino acid residues play an important role in the catalytic site. The hydrogen bond interaction (D3) with Tyr4800 facilitates the best alignment of Nε-Lys4 with the methyl group of the SAM cofactor. In contrast, the hydrogen bond interaction between the Nε-Lys4 and Tyr4886 (D4) appeared critical for the catalytic function to transfer the methyl group from the SAM cofactor to the substrate (Trievel et al., 2002).

In summary, our results demonstrate how changes in the amino acid sequence or conformational changes affect the geometry of the protein structure resulting in the loss of hydrogen bond interactions across variants. Hydrogen bonds, although individually weaker than some other intermolecular forces, collectively contribute significantly to overall protein stability (Pace, 2009; Majewski et al., 2019). They play a crucial role in aligning molecular groups in a specific orientation giving proteins a defined structure. Consequently, disturbance of these interactions can destabilize the protein structure, resulting in decreased stability and functionality (BNt et al., 2021). This information bears not only mechanistic significance but may orient future studies on drug development.

### 3.7 Integrative analyses reveal that all variants disrupt at least one of the structural or dynamics features

Lastly, we integrate the data from the structure and dynamics of KMT2C to establish a molecular fitness (MF) behavior, used it to classify variants as SV (Structural Variants), DV (Dynamic Variants), and SDV (Structural and Dynamic Variants). Our dynamics data is compiled from time-dependent interaction energies and time-dependent interaction bonds related to the SET domain’s function. The score-specific thresholds applied to dynamic scores to classify the effects of variants are described in Supplementary Table S3 (Supplementary Material).

**FIGURE 5:**
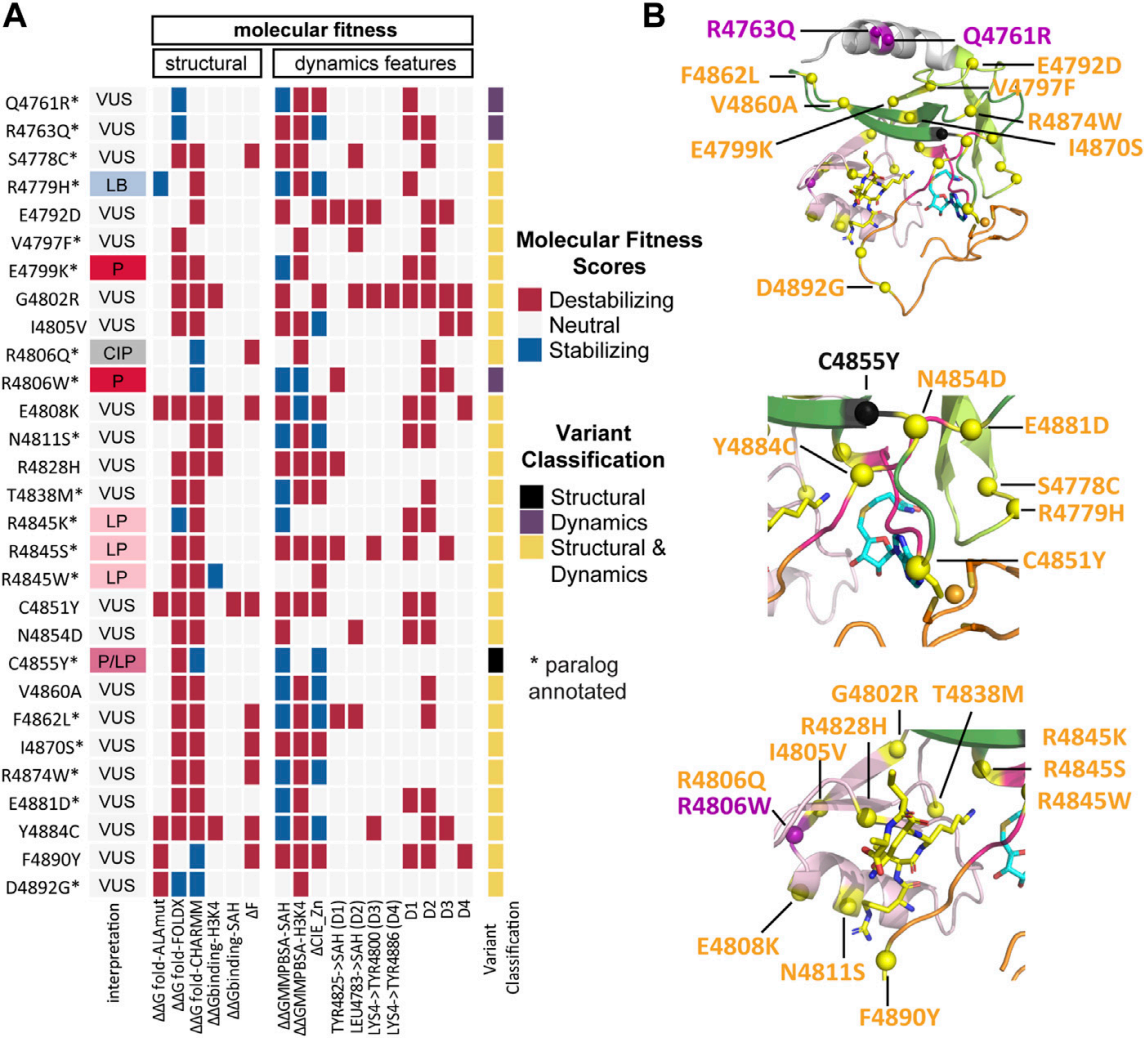
Integrative analyses reveal that all variants disrupt at least one of the structural or dynamics features. **(A)** KMT2C variant scores based on molecular fitness (MF), which is derived from the integration of data from the structure (Table 2) and dynamics features (Supplementary Table S3) and used to classify variants into SV (Structural Variants), DV (Dynamic Variants), and SDV (Structural and Dynamic Variants). The MF scoring reveals that all variants destabilize at least one of the structural or dynamics features. The interpretation column refers to ClinVar interpretation, and the variant classification column refers to the classification by our MF analysis. **(B)** Mapping of variants onto the KMT2C SET domain molecular structure. Variants are indicated as spheres, SV in black, DV in purple and SDV in yellow. (*Upper*) full view of SET Domain, *middle*) zoom-in the cofactor and Zn2+ regions, (*lower*) zoom-in the substrate catalytic site. The same color codes for the sub-domains shown in Figure 1D are used. The protein is shown in cartoon representation. The cofactor product SAH and the substrate H3K4 are shown in a stick model with carbon atoms colored in blue and yellow, respectively, oxygen in red, nitrogen in blue, and sulfur in yellow. The Zn^2+^ ion is shown as an orange sphere.

The MF scoring reveals that all variants destabilize at least one of the structural or dynamics features (Figure 5A). According to our simulations, substituting Gly for Arg at position 4,802 resulted in 12 destabilizing effects of 17 scores. Even though variant G4802R is interpreted as VUS on Clinvar, our results highlight the potential of this variant to cause dysfunction of KMT2C enzymatic activity.

MF classifies only one variant, C4855Y, as SV based on ΔΔG_fold-FoldX_ destabilizing effects. This variant is annotated as Pathogenic/Likely Pathogenic on ClinVar. The VUS Q4761R and R4763Q variants, as well as the pathogenic R4806W variant, exhibited destabilizing effects following the dynamic simulations, categorizing them as DV. The remaining 25 variants were classified as SDV, including the likely pathogenic hotspots R4845K, R4845S, and R4845W. Finally, the annotations of the 21 classified VUS have been enhanced using our method, where we aggregate valuable information related to structure and dynamics behavior (Figure 5B).

Thus, the combination of multidimensional data provides more comprehensive information about the MF of KMT2C genomic variants, which can aid in diagnosing and treating individuals carrying KLEFS-2-associated mutations.

## 4 Discussion

The current study reflects our efforts to standardize, parametrize, and innovate the application of computational biophysics, biochemistry, and chemical biology to advance methods of interpretation for disease-associated genomic variations. Thus, the data presented here from our multi-tiered approach provides knowledge on the potentially damaging effects of distinct variants and their mechanisms of dysfunction. We are applying these methodologies to study chromatinopathies, namely, diseases caused by mutations in epigenomic regulators. In this regard, published work from our laboratory has focused on the Kabuki syndrome (Chi et al., 2021). Here, we extend our investigations to related syndromes, specifically Kleefstra syndrome II. Our structural and functional analysis offers a significant opportunity to understand how germline SNVs may affect KMT2C enzymatic activity, a member of the methyltransferase family responsible for several human chromatinopathies. The Clinvar database is an important source for mining information on germline SNV in KMT2 family members. However, most variants remain classified as VUS, with conflicting interpretations and many unprovided classifications. These numbers reach an impressive three-quarters of the total and represent a significant clinical burden for diagnosed patients and their families, highlighting the necessity for enhancing the annotation to understand the role of these genes in rare diseases.

To increase the level of information, we used paralog annotation, permitting coverage of a broad spectrum of mutations in the KMT2C SET domain. The evolutionary-related gene KMT2D shares the homologous sequence and protein domain and was identified as a gene of interest. The interpretation of SNV in the conserved SET domain, it is crucial to understand the functional mechanism of KMT2C/D paralogs. The annotation of equivalent amino acids in paralog proteins provides a valuable tool for predicting the potential impact of missense variants in proteins, like KMT2C, for which there is limited clinical genetic information. Additionally, we described the use of multidimensional tools, such as 2D sequence-based scores with the protein 3D structure scores and 4D time-dependent dynamic scores, to extend our understanding of the SNV interpretation.

We initiated this study by analyzing multiple prediction algorithms to assess the pathogenicity of variants. Variant E4792D, described in the subdomain SET-C, showed less confident prediction rates. This lack of agreement can be attributed to the fact that this glutamic acid is a non-conserved residue-based on multiple sequence alignments across KMT2 family members and is an aspartic acid in the KMT2A and KMT2B proteins (Supplementary Figure S6 in Supplementary Material). In fact, the conflicting results can be ascribed to the development of a large set of tools to predict the pathogenicity of SNV based on the conservation and biochemical properties of the amino acid substitutions using different predictive features.

On the other hand, the initial structural analysis reveals that the SNV has the potential to produce a phenotypic effect by impacting the stability of the KMT2C protein and/or by impairing the activity of KMT2C without changing its stability but by blocking the KMT2C catalytic site based on alterations in the affinity with the histone substrate or SAM cofactor. Our results point out that 90% of the SNV studied here falls in at least one of the six 3D scores and may damage the function of KMT2C.

In our third level of analysis, we analyzed the MD trajectory of the models to obtain scores related to time-dependent behavior, including protein stability, conformational changes, and binding affinities. Trajectory analysis provided quantitative measures of properties to compare the molecular behavior of the different variant models to the canonical WT model.

Overall, this study highlights the importance of combining multiple approaches to understand the functional consequences of genomic variants at different information levels. We calculate and combined data from structural properties and dynamic behavior data to classify KMT2C and paralog variants. Further investigation, including MD, provides more refined data than the current annotation tools used in human genomics databases. Our built 3D models for variants presented in the SET domain regions suggest distinct mechanisms that lead to their imbalance and are likely not predictable from sequence alone.

Using *in silico*-established tools allowed us to gain insights about the consequences of SNV. Our MD simulations provide a deep understanding of disturbances in interactions that can destabilize the protein structure, resulting in decreased stability and functionality.

This new knowledge extends our understanding of the molecular mechanism underlying the dysfunction of KLEFS type 2-associated genomic mutations. We will continue pursuing new metrics to improve our multidimensional analysis and better understand how genetic variation translates to functional mechanisms. Identifying KMT2C mutation-specific druggable conformations may pave the way to developing small molecules to ameliorate the symptoms of diseases related to this protein and diseases caused by KMT2C paralogs.

## Data Availability

The original contributions presented in the study are included in the article/Supplementary Material, further inquiries can be directed to the corresponding author.
