# Supplementary material for: Deep computational phenotyping of genomic variants impacting the SET domain of KMT2C reveal molecular mechanisms for their dysfunction
Source: Front Genet. 2023 Nov 28;14:1291307. doi: 10.3389/fgene.2023.1291307 (PMC10715303; doi:10.3389/fgene.2023.1291307)
Supplement: Supplementary file 1 [file Presentation1.pdf]

Supplementary Material

Deep Computational Phenotyping of Genomic Variants Impacting the SET Domain of KMT2C Reveal Molecular Mechanism for Their Dysfunction

Salomao Doria Jorge, Young-In Chi, Jose Lizarraga Mazaba, Neshatul Haque, Jessica Wagenknecht, Brian C. Smith, Brian F. Volkman, Angela J. Mathison, Gwen Lomberk, Michael T. Zimmermann and Raul Urrutia

Table of contents

Table S1	List of pathogenicity prediction algorithms based on chromosome and position on genome version GRCh38 ……………………………………………………	S2
Figure S1	Lollipop plot of reported KMT2C and KMT2D variants ………………………….	S3
Figure S2	*In silico* Alanine mutagenesis scanning ………………………………………………	S4
Figure S3	Two-dimensional (2D) plots for WT:complexes and variants that destabilize the interaction …………………………………………………………………	S5
Figure S4	The MD simulation of 10 replicates of WT-KMT2C complex ……………………	S6
Figure S5	Superposition of time dependent RMSF plots ……………………………………	S7
Table S2	WT:KMT2C and variant scores based on molecular dynamics simulations……….	S10
Table S3	Scores and classification of KMT2C variants in the SET domain based on dynamics data ………………………………………………………………………………………….	S11
Figure S6	Multiple sequence alignment (MSA) of the SET Domain of KMT2 family protein ………………………………………………………………………………………………	S12

TABLE S1. List of pathogenicity prediction algorithms based on chromosome and position on genome version GRCh38

Algorithm	Score Range	Interpretation	Reference
SIFT	0 to 1	The smaller the score the more likely the SNP has damaging effect	10.1093/nar/gkg509
SIFT4G	0 to 1		10.1038/nprot.2015.123
Polyphen2	0.03061 to 0.91137	"D" (""probably damaging"", [0.957,1]), "P" ("possibly damaging", [0.454,0.956]) and "B" ("benign", [0,0.452])	10.1038/nmeth0410-248
FATHMM	-16.13 to 10.64	The smaller the score the more likely the SNP has damaging effect	10.1002/humu.22225
MutationAssessor	-5.17 to 6.49	The score cutoffs between "High" and "Medium", "Medium" and "Low", and "Low" and "Neutral", are 3.5, 1.935 and 0.8, respectively	10.1093/nar/gkr407
MutationTaster	0 to 1	MutationTaster predicts a variant as *deleterious* or *benign*	10.1038/nmeth.2890
MutPred	0 to 1	The larger the score the more likely the SNP has damaging effect	10.1093/bioinformatics/btp528
PROVEAN	-14 to 14	The smaller the score the more likely the SNP has damaging effect	10.1093/bioinformatics/btv195
GERP_RS	-12.3 to 6.17	The larger the score, the more conserved the site	10.1371/journal.pcbi.1001025
PhiloP30way	-20 to 1.312		10.1101/gr.097857.109
PhastCons30way	0 to 1		10.1101/gr.3715005
CADD	-6.458163 to 18.30149	The larger the score the more likely the SNP has damaging effect	10.1093/nar/gky1016
DANN	0 to 1	A larger number indicate a higher probability to be damaging	10.1093/bioinformatics/btu703
fathmm_MKL	0 to 1	SNVs with scores >0.5 are predicted to be deleterious, and those <0.5 are predicted to be neutral or benign. Scores close to 0 or 1 are with the highest confidence	10.1093/bioinformatics/btv009
fathmm_XF	0 to 1		10.1093/bioinformatics/btx536
GenoCanyon	0 to 1	SNVs with scores >0.5 are predicted to be deleterious, and those <0.5 are predicted to be neutral or benign	10.1038/srep10576
METALR	0 to 1	Larger value means the SNV is more likely to be damaging	10.1038/s41598-018-38189-9
MetaSVM	-2 to 3	Larger value means the SNV is more likely to be damaging	10.1186/s13040-017-0126-8
REVEL	0 to 1+	The larger the score the more likely the SNP has damaging effect	10.1016/j.ajhg.2016.08.016
VEST4	0 to 1	The larger the score the more likely the mutation may cause a functional change	10.1186/1471-2164-14-S3-S3

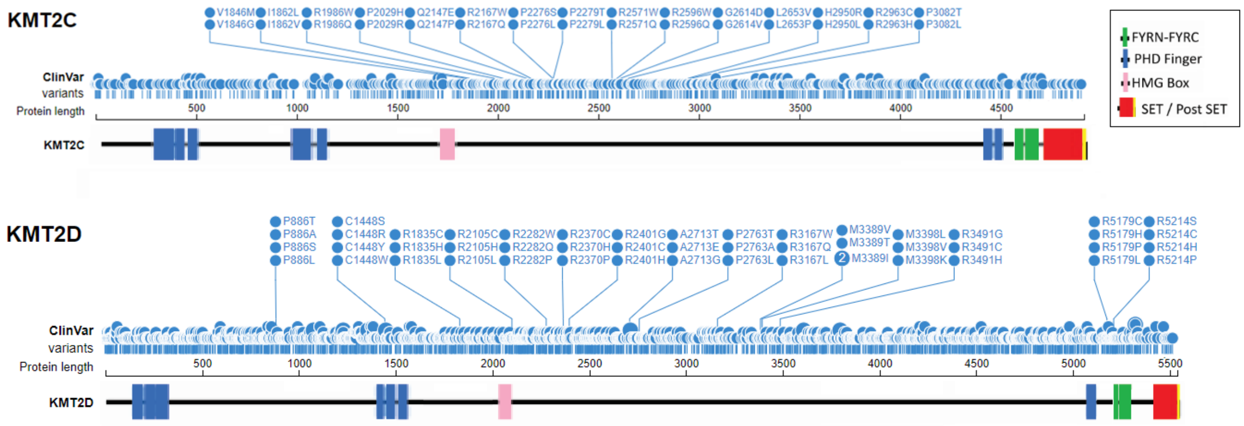

Figure S1. Lollipop plot of reported KMT2C and KMT2D variants in the literature and two-dimensional representation of their locations in the domain organization of the proteins (x-axis). FYRN/FYRC = phenylalanine and tyrosine-rich region (*N*- and *C*-terminal); HMG, high mobility group; N-SET = N-terminal of SET; PHD, plant homeodomain; Post-SET, *C*-terminal of SET; SET = Su(var)3-9.

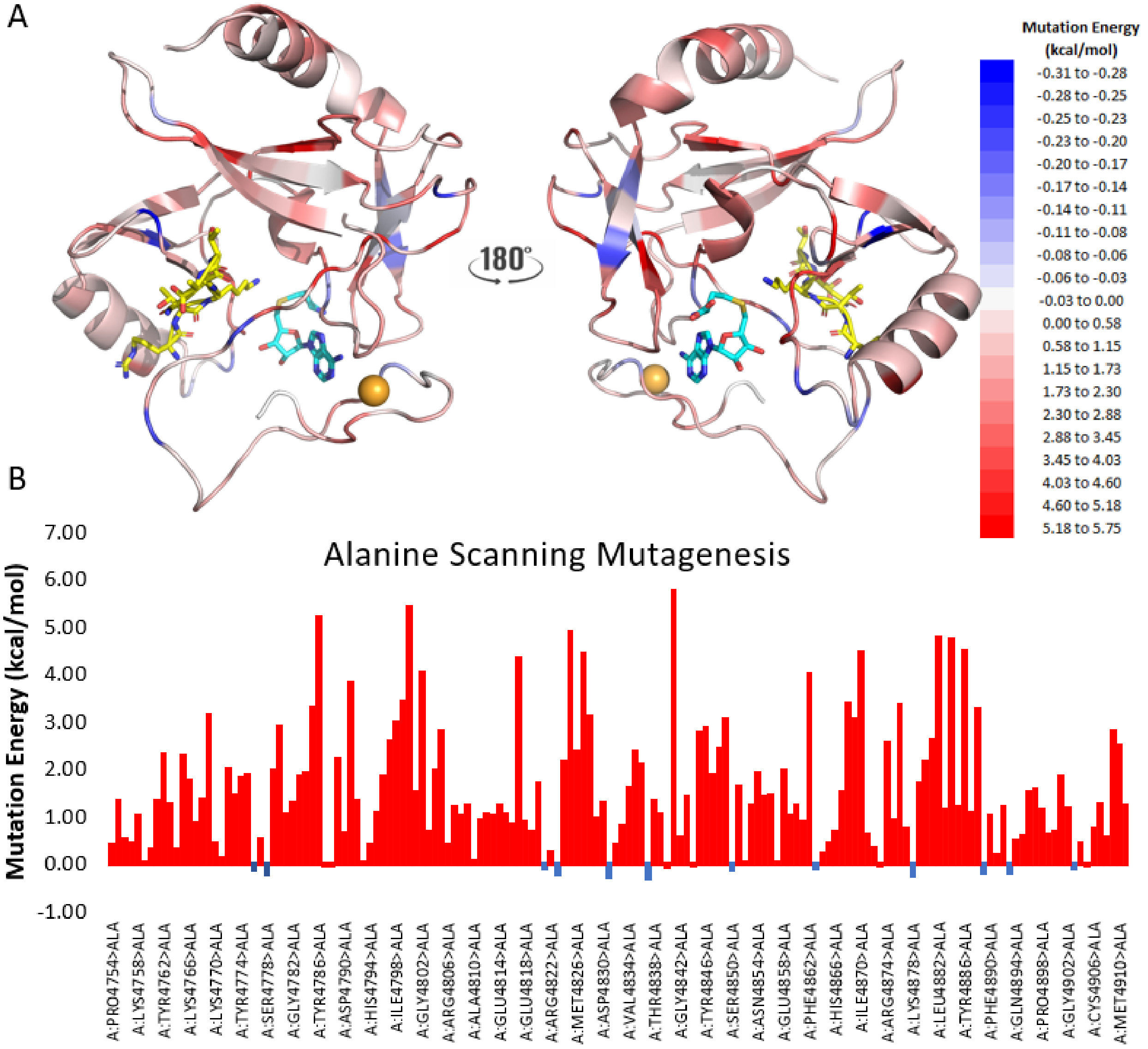

Figure S2. *In silico* Alanine mutagenesis scanning. **(A)** Heatmap of the mutation energy values projected in the WT:KMT2C complex model. Cartoon representation model of KMT2C is colored by mutation energy values. The cofactor product SAH and the substrate H3K4 are shown in stick model with carbon atoms colored in blue and yellow, respectively, oxygen in red, nitrogen in blue, and sulfur in yellow. The Zn^{2+} ion is shown as an orange sphere. **(B)** Contribution of the side chains of the residues to KMT2C function by mutating by the smaller alanine residue. For each KMT2C variant, the simulation calculates the difference between the folding free energy ($\Delta\Delta G_{fold-ALAmut}$) of the wild type and the mutated structure.

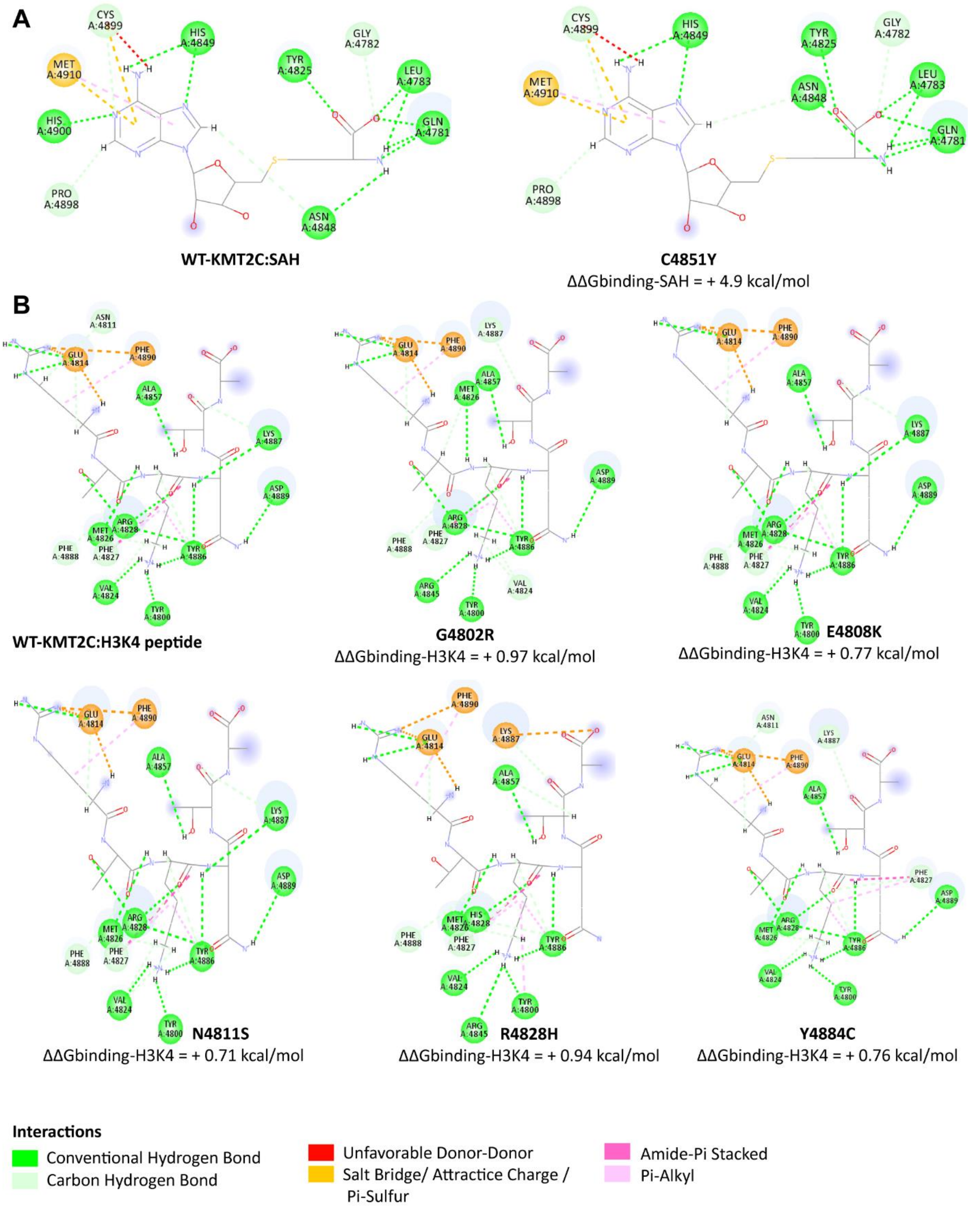

Figure S3. Two-dimensional (2D) plots for WT:complexes and variants that destabilize the interaction. **(A)** 2D interaction diagram of SAH with the WT and C4851Y variant. **(B)** 2D interaction diagram of H3K4 with the WT and G4802R, E4808K, N4811S, R4828H, and Y4884C variants.

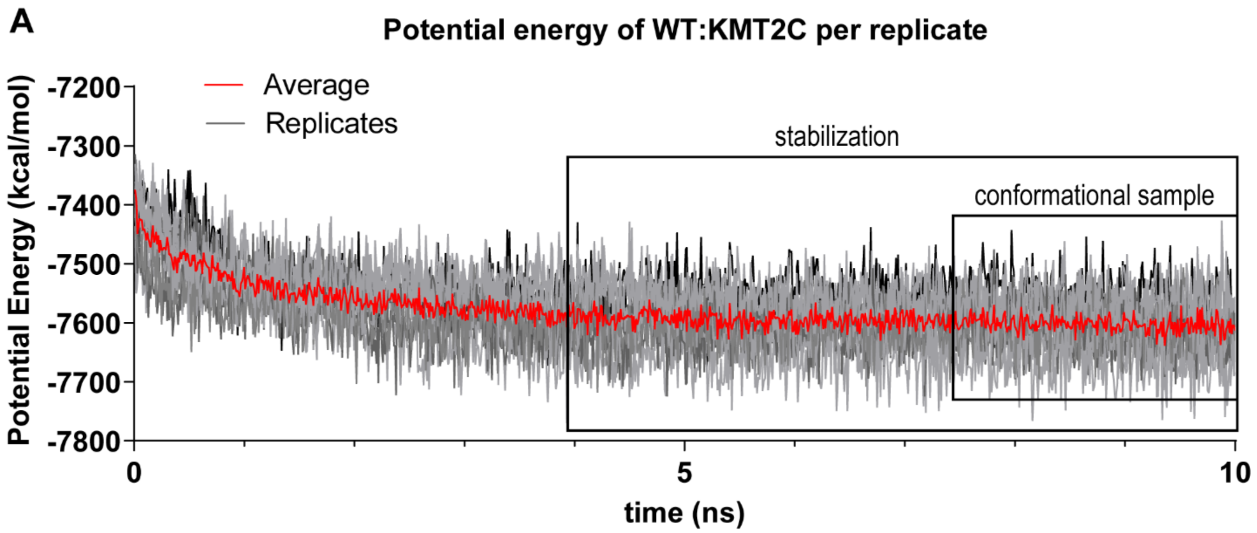

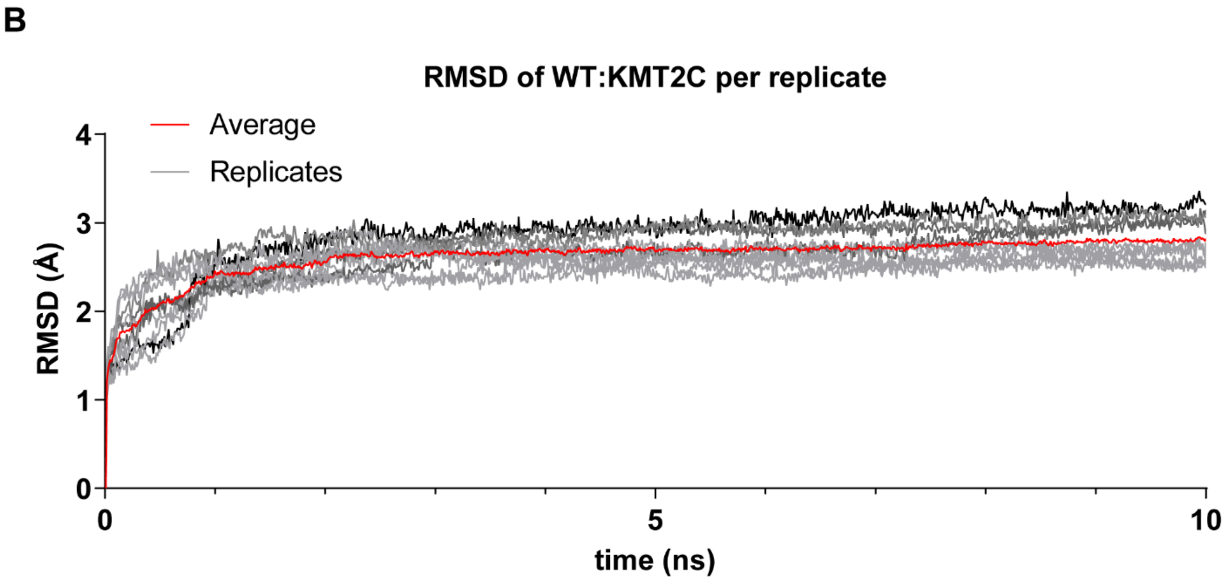

Figure S4. The MD simulation of 10 replicates of the WT:KMT2C complex. **(A)** The potential energy plot of WT:KMT2C model during MD simulation. The protein stabilizes at approximately 4 ns, with repetitive movements. To further analyze the time-dependent interactions, 250 conformations were extracted from the last 2.5 ns of each simulation. **(B)** The RMSD plot calculated for the WT-KMT2C complex explains the equilibration nature of the protein throughout the 10 ns.

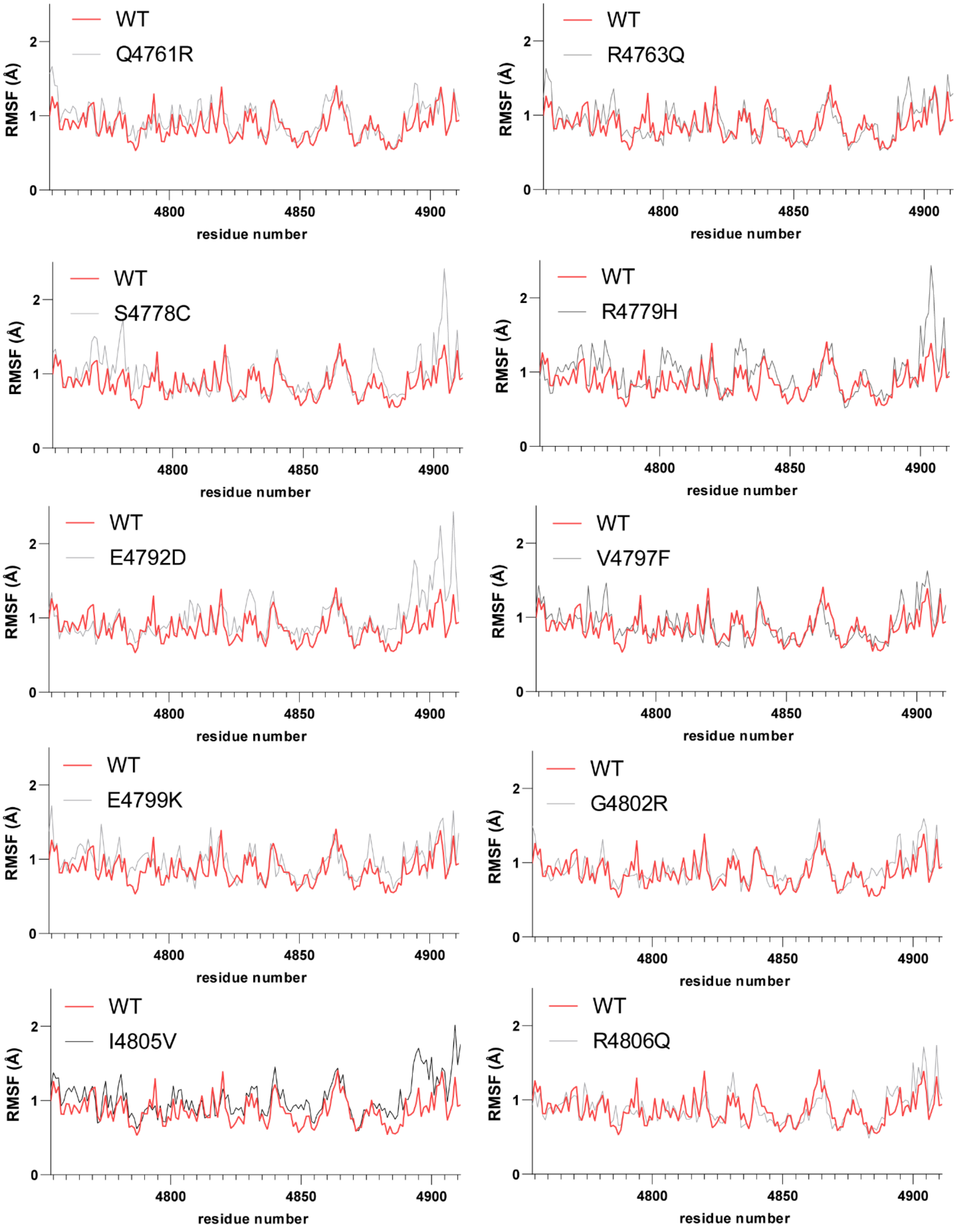
WT
Q4761R
R4763Q
S4778C
R4779H
E4792D
V4797F
E4799K
G4802R
I4805V
R4806Q
RMSF (Å)
residue number
0
1
2
4800
4850
4900

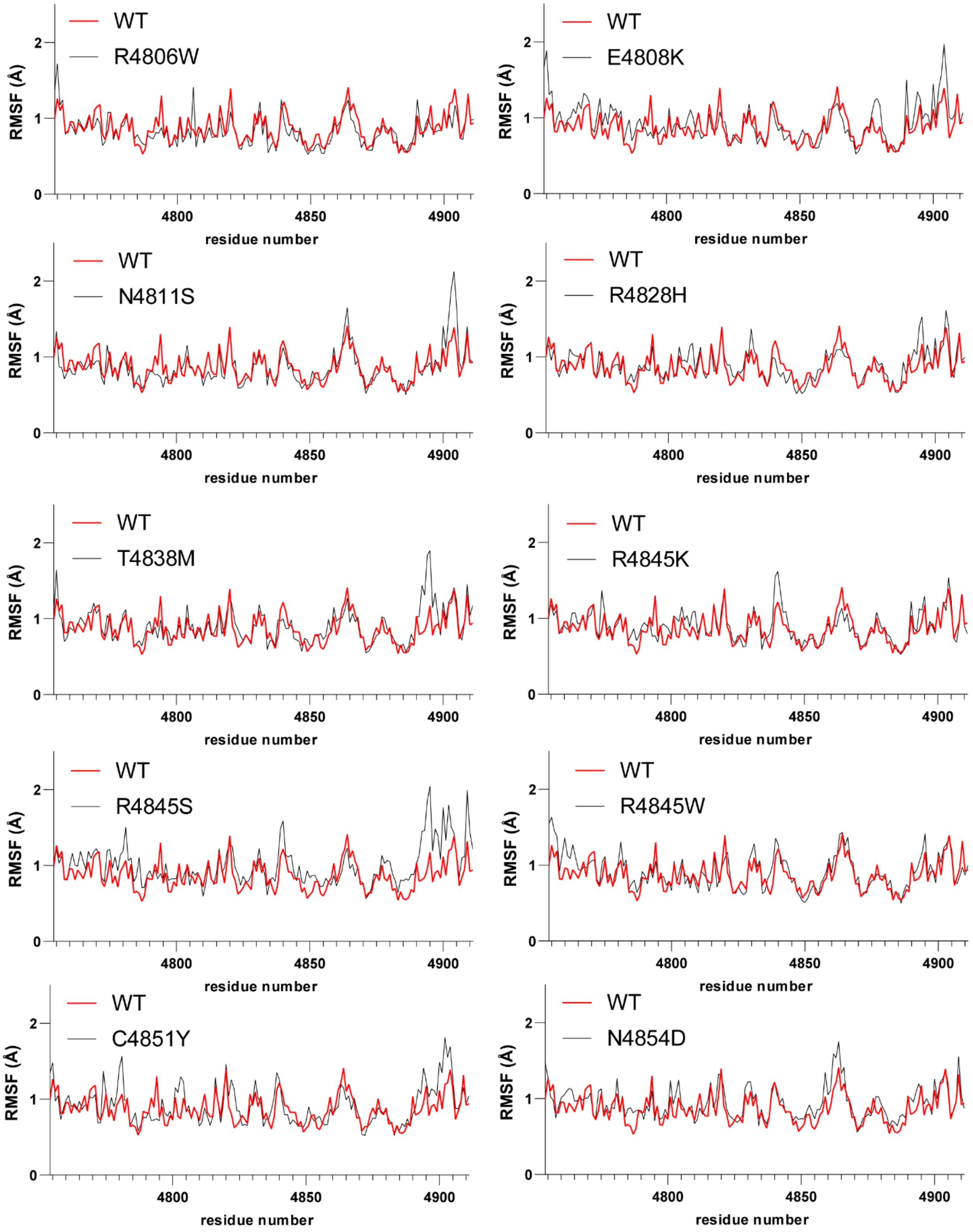
WT
R4806W
E4808K
N4811S
R4828H
T4838M
R4845K
R4845S
R4845W
C4851Y
N4854D
RMSF (Å)
residue number
0
1
2
4800
4850
4900

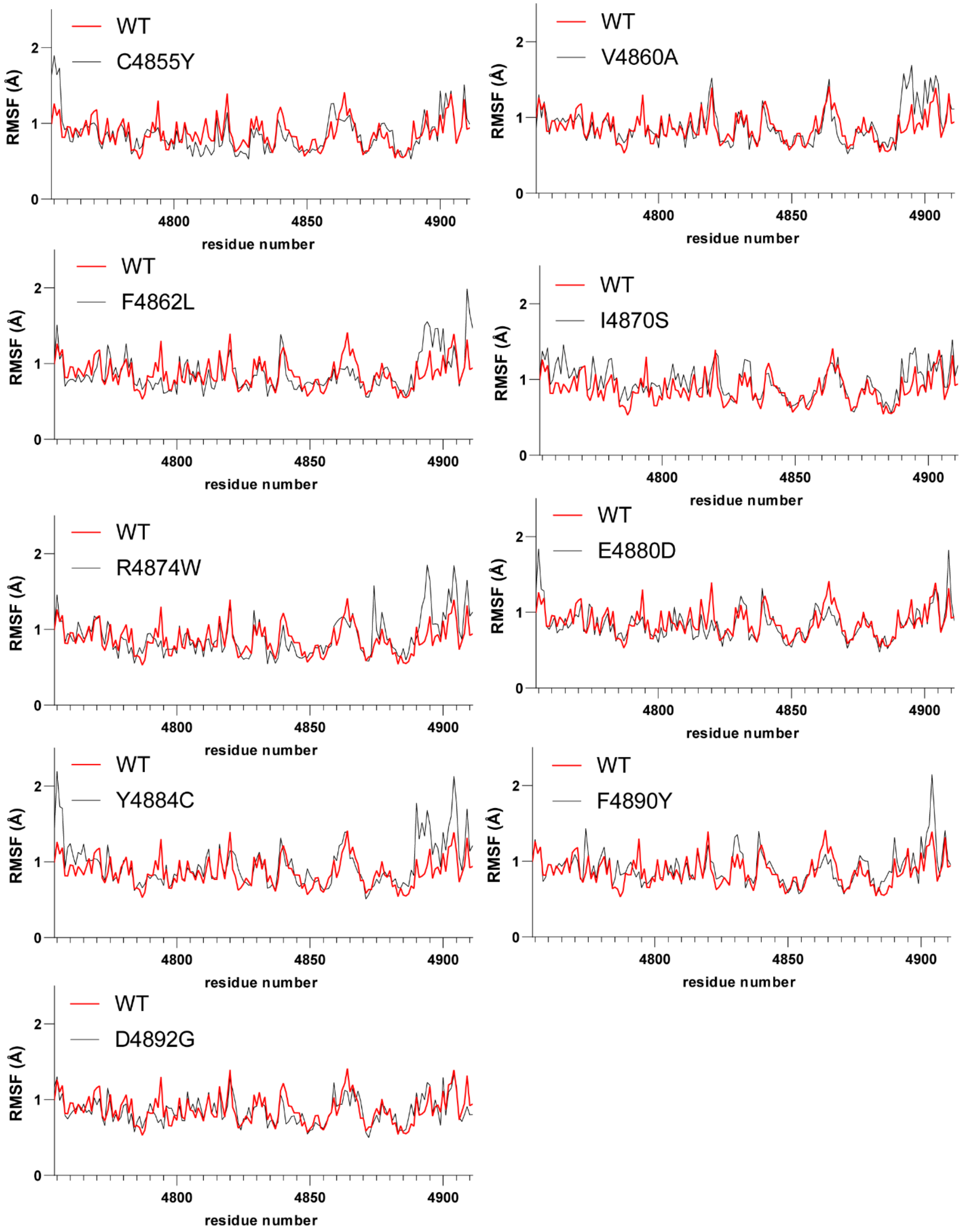

Figure S5. Superposition of time-dependent RMSF plots of the averaged values of 10 replicates of WT:KMT2C (red) and each variant (gray) per individual residues during the MD production stage.

Table S2. WT:KMT2C and variant scores based on molecular dynamics simulations.

paralog	interpretation	*RMSD*	*Rg*	*r-RMSF*	*Δ|RMSF|*	*SASA*	*MMPBSA*			*time-dependent frequency*				*distances*			
							ΔΔGSAH	*ΔΔGH3K4*	*CIE_Zn*	*TYR4825-AdoHcy (D1)*	*LEU4783-AdoHcy (D2)*	*LYS4-TYR4800 (D3)*	*LYS4-TYR4886 (D4)*	*D1*	*D2*	*D3*	*D4*
WT	WT	1.25	14.96	1	0	6777.86	-163.44	-98.04	-511.73	0.56	0.33	1	1	2.898	2.967	2.464	2.461
Q4761R*	VUS	1.32	15.14	0.76	0.04	6849.65	-169.33	-81.90	-504.44	0.5	0.47	0.76	0.39	3.606	3.221	2.484	2.969
R4763Q*	VUS	1.22	15.05	0.75	0	6742.79	-139.97	-84.55	-520.90	0.65	0.25	0.52	0.45	3.497	3.523	2.885	2.682
S4778C*	VUS	1.29	14.95	0.75	0.02	6829.1	-155.44	-81.46	-511.32	0.66	0.08	0.6	0.55	2.813	6.534	3.019	2.845
R4779H*	LB	1.24	15.04	0.68	0.02	6784.19	-193.17	-73.92	-524.75	0.6	0.45	0.52	0.8	3.991	3.175	2.845	2.569
E4792D	VUS	1.21	14.9	0.7	0.01	6627.33	-132.79	-96.39	-502.01	0.19	0.1	0.1	0.38	3.052	4.335	8.075	3.206
V4797F*	VUS	1.22	14.98	0.84	0	6857.27	-166.77	-86.33	-512.60	0.8	0.07	0.52	0.33	3.111	6.126	2.614	2.678
E4799K*	P	1.29	14.96	0.84	0.06	6583.5	-175.18	-87.00	-516.62	0.76	0.37	0.52	0.47	4.558	4.389	2.886	2.761
G4802R	VUS	1.41	14.91	0.82	0.09	6845.95	-151.28	-93.40	-506.58	0.23	0	0	0.11	3.658	7.112	7.558	6.773
I4805V	VUS	1.28	14.91	0.76	0.03	6836.09	-157.78	-91.78	-516.76	0.2	0.75	0.57	0.49	3.226	2.966	3.538	3.545
R4806Q*	CIP	1.2	14.92	0.77	0.01	6755.04	-165.05	-91.28	-514.00	0.47	0.49	0.62	0.48	2.758	3.605	3.177	2.802
R4806W*	P	1.18	17.97	0.83	0.03	6727.27	-181.29	-103.10	-511.26	0.19	0.22	0.24	0.24	3.148	5.08	3.649	3.17
E4808K	VUS	1.28	14.99	0.81	0.02	6749.88	-144.52	-104.57	-503.02	0.75	0.22	0.62	0.32	4.126	3.475	3.072	5.125
N4811S*	VUS	1.14	15	0.85	0.04	6775.49	-183.23	-87.71	-518.64	0.77	0.37	0.36	0.43	3.734	3.598	3.397	2.793
R4828H	VUS	1.27	15.06	0.69	0.02	6781.69	-151.57	-83.20	-501.88	0.19	0.84	0.36	0.56	2.35	2.827	3.16	2.668
T4838M*	VUS	1.25	15.04	0.71	0.02	7041.27	-173.99	-83.64	-502.63	0.25	0.57	0.67	0.67	2.887	3.491	2.629	2.484
R4845K*	LP	1.27	14.85	0.76	0.01	6783.64	-189.99	-98.47	-507.51	0.41	0.37	0.29	0.31	4.498	5.304	3.097	3.223
R4845S*	LP	1.4	14.99	0.66	0.08	6860.06	-141.40	-91.54	-501.08	0.14	0.83	0.14	0.43	4.277	2.745	4.547	3.093
R4845W*	LP	1.16	14.84	0.75	0.02	6786.28	-163.02	-93.22	-505.01	0.72	0.42	0.9	0.79	2.844	3.131	2.945	2.497
C4851Y	VUS	1.24	15.19	0.72	0.01	6805.62	-136.72	-74.46	-491.84	0.64	0.34	0.24	0.57	4.45	3.532	3.301	2.641
N4854D	VUS	1.22	14.97	0.8	0.01	6744.04	-152.37	-95.08	-507.47	0.6	0	0.67	0.68	4.75	4.204	2.852	2.726
C4855Y*	P/LP	1.25	15.03	0.67	0.01	6891.79	-183.65	-93.13	-519.05	1	1	0.81	0.67	2.986	2.704	2.735	2.68
V4860A	VUS	1.23	15.09	0.73	0.01	6667.82	-173.56	-91.56	-525.54	0.58	0.53	0.67	0.74	2.763	5.092	2.743	2.66
F4862L*	VUS	1.21	15.06	0.65	0.01	6779.94	-174.34	-85.26	-517.25	0.11	0.02	0.9	0.64	2.875	6.341	2.601	2.723
I4870S*	VUS	1.23	14.9	0.72	0	6722.88	-153.94	-92.73	-506.01	0.49	0.56	0.38	0.46	3.201	3.055	3.304	2.848
R4874W*	VUS	1.22	15	0.73	0.04	6813.9	-175.96	-80.60	-519.69	0.41	0.7	0.79	0.33	2.713	2.553	2.565	2.744
E4881D*	VUS	1.15	15	0.8	0.02	6181.34	-170.92	-85.92	-508.05	0.51	0.23	0.69	0.76	3.798	5.737	2.634	2.597
Y4884C	VUS	1.26	15.04	0.84	0.02	6825.43	-177.60	-92.42	-523.15	0.73	0.31	0.1	0.41	2.573	4.171	5.022	2.782
F4890Y	VUS	1.26	14.89	0.69	0.01	6812.27	-142.58	-88.51	-503.16	0.52	0.29	0.36	0.23	4.061	5.004	3.178	3.963
D4892G*	VUS	1.24	14.98	0.78	0.03	6683.83	-163.92	-78.70	-512.82	0.51	0.58	0.9	0.69	2.666	3.222	2.546	2.578

* paralog annotation

Table S3. Scores and classification of KMT2C variants in the SET domain based on dynamics data

	MMPBSA [1]		*CIE_Zn* [1]	*time-dependent frequency* [2]				*distances* [3]			
	ΔΔGSAH	*ΔΔGH3K4*		*TYR4825-AdoHcy (D1)*	*LEU4783-AdoHcy (D2)*	*LYS4-TYR4800 (D3)*	*LYS4-TYR4886 (D4)*	*D1*	*D2*	*D3*	*D4*
Q4761R*	-5.89	16.14	7.29	0.50	0.47	0.76	0.39	3.61	3.22	2.48	2.97
R4763Q*	23.47	13.49	-9.17	0.65	0.25	0.52	0.45	3.50	3.52	2.89	2.68
S4778C*	8.00	16.58	0.41	0.66	0.08	0.60	0.55	2.81	6.53	3.02	2.85
R4779H*	-29.73	24.12	-13.02	0.60	0.45	0.52	0.80	3.99	3.18	2.85	2.57
E4792D	30.65	1.65	9.72	0.19	0.10	0.10	0.38	3.05	4.34	8.08	3.21
V4797F*	-3.33	11.71	-0.87	0.80	0.07	0.52	0.33	3.11	6.13	2.61	2.68
E4799K*	-11.74	11.04	-4.89	0.76	0.37	0.52	0.47	4.56	4.39	2.89	2.76
G4802R	12.16	4.64	5.15	0.23	0.00	0.00	0.11	3.66	7.11	7.56	6.77
14805V	5.66	6.26	-5.03	0.20	0.75	0.57	0.49	3.23	2.97	3.54	3.55
R4806Q*	-1.61	6.76	-2.27	0.47	0.49	0.62	0.48	2.76	3.61	3.18	2.80
R4806W*	-17.85	-5.06	0.48	0.19	0.22	0.24	0.24	3.15	5.08	3.65	3.17
E4808K	18.92	-6.53	8.71	0.75	0.22	0.62	0.32	4.13	3.48	3.07	5.13
N4811S*	-19.79	10.33	-6.91	0.77	0.37	0.36	0.43	3.73	3.60	3.40	2.79
R4828H	11.87	14.84	9.85	0.19	0.84	0.36	0.56	2.35	2.83	3.16	2.67
T4838M*	-10.55	14.40	9.10	0.25	0.57	0.67	0.67	2.89	3.49	2.63	2.48
R4845K*	-26.55	-0.43	4.22	0.41	0.37	0.29	0.31	4.50	5.30	3.10	3.22
R4845S*	22.04	6.50	10.65	0.14	0.83	0.14	0.43	4.28	2.75	4.55	3.09
R4845W*	0.42	4.82	6.72	0.72	0.42	0.90	0.79	2.84	3.13	2.95	2.50
C4851Y	26.72	23.58	19.90	0.64	0.34	0.24	0.57	4.45	3.53	3.30	2.64
N4854D	11.07	2.96	4.26	0.60	0.00	0.67	0.68	4.75	4.20	2.85	2.73
C4855Y*	-20.21	4.91	-7.32	1.00	1.00	0.81	0.67	2.99	2.70	2.74	2.68
V4860A	-10.12	6.48	-13.81	0.58	0.53	0.67	0.74	2.76	5.09	2.74	2.66
F4862L*	-10.90	12.78	-5.52	0.11	0.02	0.90	0.64	2.88	6.34	2.60	2.72
14870S*	9.50	5.31	5.72	0.49	0.56	0.38	0.46	3.20	3.06	3.30	2.85
R4874W*	-12.52	17.44	-7.96	0.41	0.70	0.79	0.33	2.71	2.55	2.57	2.74
E4881D*	-7.48	12.12	3.68	0.51	0.23	0.69	0.76	3.80	5.74	2.63	2.60
Y4884C	-14.16	5.62	-11.42	0.73	0.31	0.10	0.41	2.57	4.17	5.02	2.78
F4890Y	20.86	9.53	8.57	0.52	0.29	0.36	0.23	4.06	5.00	3.18	3.96
D4892G*	-0.48	19.34	-1.09	0.51	0.58	0.90	0.69	2.67	3.22	2.55	2.58

Destabilizing
Neutral
Stabilizing

* paralog annotation

[1] Values of ΔΔG (or CIE) > 5.0 kcal/mol classifies the effect of variant as destabilizing (loss of binding energy), whereas variants with values of ΔΔG (or CIE) < -5.0 kcal/mol classifies as stabilizing effect (increase of binding energy), and variants with neutral effects yield values of ΔΔG (or CIE) ranging from -5.0 to 5.0 kcal/mol. [2] Values of frequency < 0.20 (frequency of 20% of interaction) classifies the effect of residue as destabilizing. [3] Values of HB distance > 3.3Å classifies the effect of residue as destabilizing.

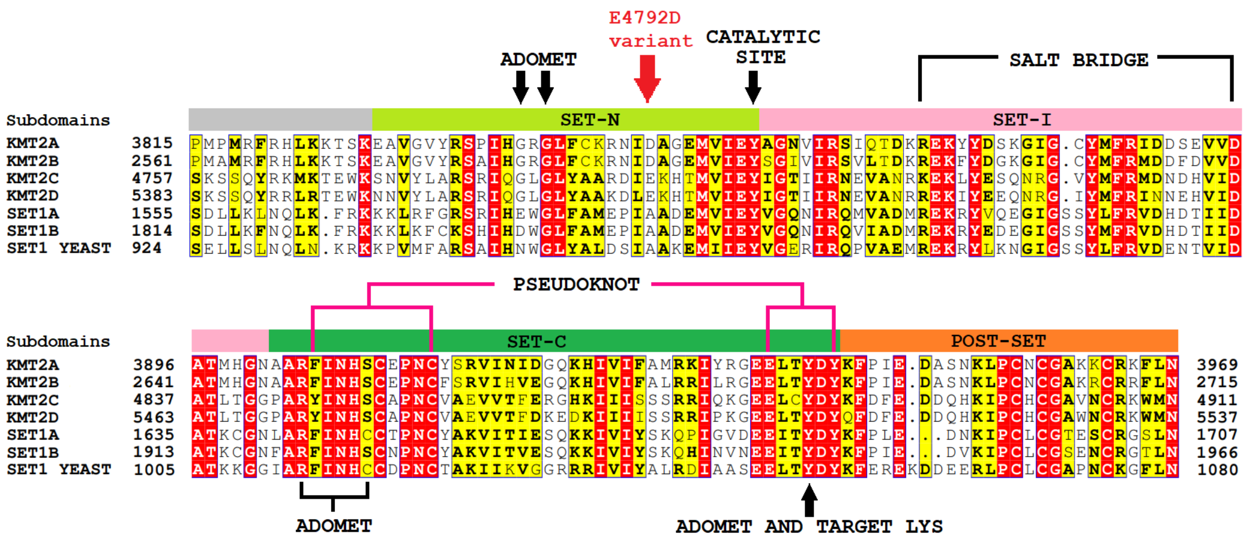

Figure S6. Multiple sequence alignment (MSA) of the SET domain of KMT2 family proteins. Positions identical between the orthologs are highlighted with a red background, and similar residues are written with bold black characters and boxed in yellow. Alignment was performed using the MultAlin server (Sievers, Wilm et al. 2011) and displayed using Esprit 3.0 Server (Robert and Gouet 2014). Above the MSA is the schematic-colored representation of subdomains of the SET domain. The residues binding to the cofactor, substrate, and salt bridge are indicated with black arrows and brackets, and the structural pseudoknot as pink brackets. Variant E4792D is indicated in red.
